# Transcriptional and Bioinformatic Analysis Provide a Relationship between Host Response Changes to Marek's Disease Viruses Infection and an Integrated Long Terminal Repeat

**DOI:** 10.3389/fcimb.2016.00046

**Published:** 2016-04-26

**Authors:** Ning Cui, Xianyao Li, Cuiying Chen, Haiyu Hao, Shuai Su, Zhizhong Cui

**Affiliations:** ^1^Department of Preventive Veterinary Medicine, College of Veterinary Medicine, Shandong Agricultural UniversityTai'an, China; ^2^Shandong Provincial Key Laboratory of Animal Biotechnology and Disease Control and Prevention, Shandong Agricultural UniversityTai'an, China; ^3^Department of Animal Nutrition and Feed Science, College of Animal Science, South China Agricultural UniversityGuangzhou, China; ^4^Qingdao Animal Husbandry and Veterinary Research InstituteQingdao, China

**Keywords:** Marek's disease virus, microarrays, bioinformatic analysis, long terminal repeat, virus evolution

## Abstract

GX0101, Marek's disease virus (MDV) strain with a long terminal repeat (*LTR*) insert of reticuloendotheliosis virus (REV), was isolated from CVI988/Rispens vaccinated birds showing tumors. We have constructed a *LTR* deleted strain GX0101Δ*LTR* in our previous study. To compare the host responses to GX0101 and GX0101Δ*LTR*, chicken embryo fibroblasts (CEF) cells were infected with two MDV strains and a gene-chip containing chicken genome was employed to examine gene transcription changes in host cells in the present study. Of the 42,368 chicken transcripts on the chip, there were 2199 genes that differentially expressed in CEF infected with GX0101 compared to GX0101Δ*LTR* significantly. Differentially expressed genes were distributed to 25 possible gene networks according to their intermolecular connections and were annotated to 56 pathways. The insertion of REV *LTR* showed the greatest influence on cancer formation and metastasis, followed with immune changes, atherosclerosis, and nervous system disorders in MDV-infected CEF cells. Based on these bio functions, GX0101 infection was predicated with a greater growth and survival inhibition but lower oncogenicity in chickens than GX0101Δ*LTR*, at least in the acute phase of infection. In summary, the insertion of REV *LTR* altered the expression of host genes in response to MDV infection, possibly resulting in novel phenotypic properties in chickens. Our study has provided the evidence of retroviral insertional changes of host responses to herpesvirus infection for the first time, which will promote to elucidation of the possible relationship between the *LTR* insertion and the observed phenotypes.

## Introduction

Marek's disease (MD) is caused by MDV and characterized by bursal-thymic atrophy with several distinct pathologic syndromes that include early mortality syndrome, cytolytic infection, immunodepression, transient paralysis, persistent neurological diseases, atherosclerosis, local lesions, and transplants in chickens (Calnek, 2001). It has long been recognized as a model for the evolution of pathogen virulence derived by vaccine (Gandon et al., 2001; Witter, 2001).

Vaccination for MD is an outstanding example of successful disease control in the area of veterinary medicine. However, frequent outbreaks of MD occur on individual farms or regions in recent years. Several reports lend credence to the implication of exceptionally virulent MDV isolates in vaccine failures (Witter et al., 1980; Powell and Lombardini, 1986; Suresh et al., 2015; Cui et al., 2016). The MDV field strain GX0101 with a long terminal repeat (*LTR*) region of reticuloendotheliosis virus (REV) insert was isolated from CVI988/Rispens vaccinated birds showing tumors in 2005 (Zhang and Cui, 2005). The pathogenesis of GX0101 was higher than virulent MDV (vMDV) GA strain but lower than very virulent MDV (vvMDV) strain Md5. The outbreak of MD caused by GX0101 was not attributed to a greater virulence but might associated with higher transmission capacity (Sun A. J. et al., 2010). It has also been demonstrated that the REV *LTR* could be integrated into the MDV genome following both long- and short-term co-infections of chicken or duck embryo fibroblasts (DEF) (Isfort et al., 1992). RM1 with a REV *LTR* insert was attenuated for oncogenicity but not for its immunosuppressive effect or *in vivo* replication (Witter et al., 1997). Several possible consequences of the REV *LTR* insertion into herpesviruses, including the transmission of retroviral information by herpesviruses, the activation, or inactivation of herpesvirus genes, the alteration of herpesvirus biological properties, etc., have also been discussed (Isfort et al., 1994; Jones et al., 1996; Witter et al., 1997). But the mechanism for the differential pathogenesis caused by the herpesviruses with/without a REV *LTR* is complex and not fully understood.

With the completion of the chicken genome sequencing and annotation endeavor and the availability of upgrade chicken-specific microarrays, gene expression profiling has become a valuable tool for evaluating host–pathogen interactions and was extensively applied to study the host response to MDV. Global gene expression profiling has been used to identify genes that are differentially expressed in response to MDV infection (Chen et al., 2011), vaccination with MD vaccines (Morgan et al., 2001), and between MD-resistant and -susceptible chicken lines (Sarson et al., 2008). Numerous genes involved in host response to MDV infection were defined in the microarray studies, which take the place to explore the mechanism involving MDV pathogenesis from the aspect of tumorigenesis, immunity, and host susceptibility. While this article has focused on the influence of insertion of REV *LTR* on global gene expression profiling of MDV-infected cells. The gene transcription profile of CEF cells infected with different MDV strains (with/without a REV *LTR*) reported here provides a unique opportunity to identify possible genes involved in the mechanism of the differential virulence of MDVs. Most significantly, this study provides an initial lead to explore the possible mechanism of emerging MDVs from vaccinated chicken flocks.

## Materials and methods

### Cell culture and MDV infection

Ten-day-old specific-pathogen free white leghorn chicken embryos for preparation of chicken embryo fibroblast (CEF) cultures were from SPAFAS Co. (Jinan, China). Primary CEF cells collected from one embryo were seeded onto three individual flasks with the density of 5 × 10^6^ cells/flask. Cells in one of the flasks were infected by GX0101 (1.5 × 10^5^ PFU/flask), the other flask infected by GX0101 Δ*LTR* (1.5 × 10^5^ PFU/flask), and the last one was mock infected as control. In total, four chicken embryos as experimental replicates were used in the current study.

### Total RNA extraction

Total RNA was extracted from GX0101-infected and GX0101Δ*LTR*-infected CEF cells at 56 h post infection by using TRIzol Reagent (Life Technologies, Carlsbad, CA, US) following the manufacturer's instructions. RNA integrity was checked on an Agilent Bioanalyzer 2100 (Agilent Technologies, Santa Clara, CA, USA). The total RNA was then purified using an RNeasy Mini Kit (QIAGEN, GmBH, Germany) and an RNase-Free DNase Set (QIAGEN, GmBH, Germany). The RNA quality was assessed by formaldehyde agarose gel electrophoresis and was quantified spectrophotometrically. High-quality RNA with an A260: A280 ratio between 1.8 and 2.0, and intact ribosomal 28S and 18S bands were used for microarray and real-time-qPCR (RT-qPCR) analysis.

### Probe labeling and microarray hybridization

A 4 × 44 K Agilent custom chicken oligo microarray (array ID: 042688) was used to compare transcription profile of GX0101-infected and GX0101Δ*LTR*-infected CEF cells. Four biological replicates were used in each group with dye balance. Fluorescently labeled complementary RNA (cRNA) probes were generated by using the Two Color Microarray Quick Labeling kit (Agilent Technologies, Santa Clara, CA, USA) and following the manufacturer's instructions. Labeled cRNA were purified by RNeasy mini kit (QIAGEN, GmBH, Germany). Each slide on the 4 × 44 K microarray was hybridized with 825 ng Cy3-labeled cRNA and 825 ng Cy5-labeled cRNA using Gene Expression Hybridization Kit (Agilent Technologies, Santa Clara, CA, USA) in Hybridization Oven (Agilent Technologies, Santa Clara, CA, USA), according to the manufacturer's instructions. After 17 h hybridization, slides were washed in staining dishes (Thermo Shandon, Waltham, MA, USA) with Gene Expression wash Buffer Kit (Agilent Technologies, Santa Clara, CA, USA). The labeling, hybridization and washing procedures were followed according to Agilent's recommendation and described in detail previously (Chiang et al., 2008). Slides were scanned by Agilent Microarry Scanner (Cat#G2565CA, Agilent technologies, Santa Clara, CA, US) and Feature Extraction software 10.7 (Agilent technologies, Santa Clara, CA, US).

### Microarray data normalization and analysis

Signal intensity of each probe was filtered against negative controls before normalization. Comparisons were made between CEF cells infected by MDV GX0101 and CEF cells infected by GX0101Δ*LTR*. Data normalization was performed using locally weighted scatter plot smoothing (LOWESS) by R project (http://www.r-project.org). The normalized natural log intensities were analyzed by SAS using a mixed model (SAS, Cary, NC) with fixed effect of infection with MDV (GX0101 or GX0101Δ*LTR*) and dye (Cy5 or Cy3), interaction between CEF cells and treatment, and random effect of slide and array. *P*-value and relative fold changes between each comparison for each gene were calculated. After analysis using the method, a gene was considered to be significantly differentially expressed only if the log_2_ median of the ratios of the Cy5: Cy3 signal was greater than 1.00-fold or lower than −1.00-fold with *P* ≤ 0.05.

### Ingenuity pathway analysis

Functional interpretation of significantly differentially expressed genes were analyzed in the context of gene ontology and molecular networks by using Ingenuity Pathway Analysis (IPA) software (Ingenuity Systems; www.ingenuity.com). Those genes with known gene-probe ID numbers and corresponding expression fold-changes were uploaded into the software. In IPA, the analysis was done with *P* ≤ 0.05 as the cut-off point. The IPA analysis assigned those genes into subcategories within each category, and supplied an appropriate *P*-value and the number of genes identified. The differentially expressed genes were also mapped to genetic networks in the IPA database and ranked by scores that denoted the probability that a collection of genes equal to or greater than the number in a network could be achieved by chance alone. IPA uses a right-tailed Fisher's exact test to calculate the *P*-value for functional categories, networks, and canonical pathway analysis.

### Confirmation of microarray results by RT-qPCR

Validation of differential gene expression was performed for a number of genes that were found to be differentially expressed in the microarray analysis by RT-qPCR. The primers (Table 1) were designed by using the PRIMER3 program and were based on published target sequences. RT-qPCR was performed using a 7500 System (ABI, Singapore) with SYBR®; Premix Ex Taq™II (TaKaRa, China). The temperature program used for the amplification was 95°C for 30 s and 40 cycles of 95°C for 5 s, and 60°C for 34 s. Glyceraldehyde-3-phosphate dehydrogenase (*GAPDH*) was used as the endogenous reference gene. The relative fold change of the differentially expressed genes was calculated through the 2^−ΔΔCq^ method (Livak and Schmittgen, 2001). Triplicate RT-qPCRs were performed on each cDNA to guarantee the reproducibility of the amplification.

**Table 1:** **Validation of microarray data by real-time RT-qPCR**.

**Gene symbol**	**Primer sequences (5′–3′)**	**Fold change**	
		**Microarray analysis**	**Real-time RT-qPCR**
*RGS3*	F: AGCACACCAAGGAGAACCTG	2.08	1.87
	R: ACAAGTCAGAGCGGAGGAAG		
*WBP2*	F: CCCACTTTGGCTGAATCCT	2.20	2.36
	R: ATCTCTCCCCTTGCTCTGCT		
*IFNGR2*	F: GTGGAATTTGAAGGCGAAATG	36.88	13.33
	R: GGGAGGAGGAGGGAATAGGA		
*CNTN4*	F: GAGGAGAGCAGATGGGAAAC	2.18	1.74
	R: TTCAGCGACACACTCGTAGA		
*CD83*	F: GAGTCTAAGATGACTGTGGATTTT	5.00	3.58
	R: GGCTGGCTTGTTTCGTAGAGTT		
*IRF1*	F: CTCACACACAGGGCACCAA	2.05	1.94
	R: GAGCAGCAACCAACAGAGACC		
*EPHA2*	F: AAGGGTGCTGGAGGATGAC	3.14	3.26
	R: AGAGGTGAACTTGCGGTATGA		
*CAB39*	F: AATCGCACAAAAATCCTGCT	0.43	0.42
	R: GACACTTCCTCTGATGCCTTG		
*IL-4*	F: GGTTTCCTGCGTCAAGATGAAC	0.31	0.37
	R: GTGCTGGCTCTCCCAAACA		
*CHRNG*	F: GCAGGAACCAGGAGGAGAA	0.69	0.52
	R: GAGGGTGAGTTTGAGGGTGA		
*NRXN1*	F: CAGGCATTGGACACGCTAT	0.23	0.39
	R: AAGTCATCAGAGCCCAGCAT		
*MMP11*	F: TGAAAGTCTGGAGCGATGTG	0.71	0.65
	R: TTGGGGAAGAATGCGTGT		
*COL1A2*	F: GCAGCGGTTTCTACTGGATT	0.38	0.44
	R: TTTTGTCCTTGGGGTTCTTG		
*GJA1*	F: AGAGCACGGCAAGGTAAAGA	0.32	0.43
	R: GCACTCAGGCTAAACCCATAA		
*FGF7*	F: CCCTGAGCGACATACCAGA	0.22	0.27
	R: CTGCCACTGTTCGGATTTCTA		

## Results

### Gene transcription changes in host cells infected with different MDVs

In order to characterize the relationship between host response changes to MDV infection and an integrated *LTR* further, microarray analysis with GX0101-infected, and GX0101Δ*LTR*-infected CEF cells were performed. The infected cells for microarray analysis were collected at 56 h post-infection, based on the transform status (defined MDV specific plaques). Transcription level of 2199 normalized genes were significantly altered in the GX0101-infected cells during the transformation stage, as compared to the GX0101Δ*LTR*-infected cells (shown in Supplementary Table). The insertion of *LTR* caused 720 up-regulated genes and 1479 down-regulated genes in MDV-infected cells.

### Validation of differential transcripts

RT-qPCR was used to verify a subset of 15 differential expressed genes between GX0101-infected and GX0101Δ*LTR*-infected cells during the transform stage of MDV infection. We found that the RT-qPCR results of the 15 selective genes were consistent with the microarray analysis (Table 1).

### Annotation of the differentially expressed genes

**Figure 1:**
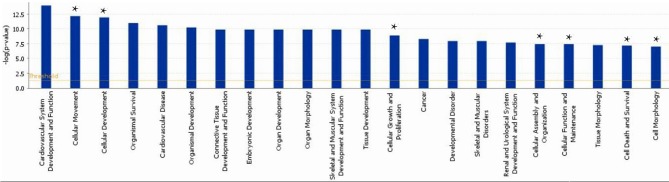
**Functional gene ontology for differentially expressed genes**. The top 16 functional groups, with larger numbers of genes, were categorized by using the IPA program. ^*^indicated the top molecular and cellular functions.

**Figure 2:**
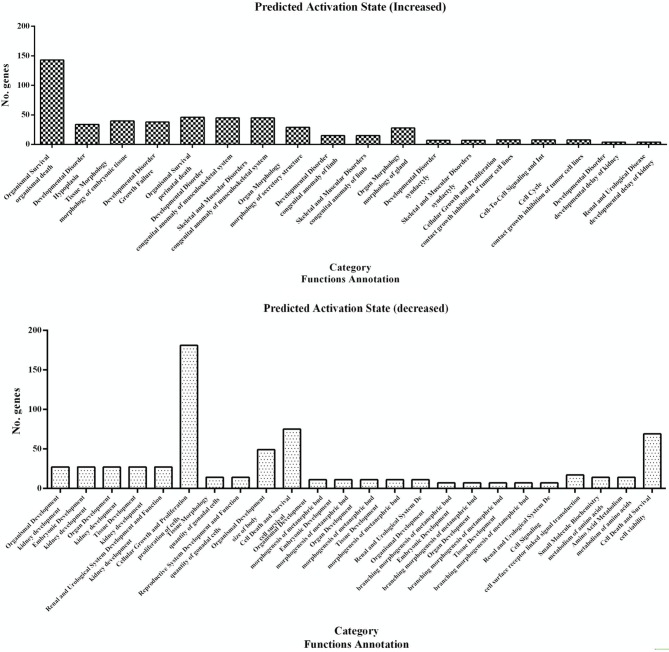
**Predicted activation state of 460 differentially expressed genes analyzed with the IPA program**. Important focus molecules belonging to different category were displayed according to their function annotation. Predicted activation state (increased/decreased) are presented according to *p*-value.

A subset of 460 differentially expressed genes have been characterized with specific bio functions in IPA. The bio function of other genes remain to be determined. The 460 differentially expressed genes were distributed to three categories including molecular and cellular functions, disorders and diseases, physiological responses, and physiological system development. It can be seen that the top molecular and cellular functions were cellular movement, cellular development, cellular growth and proliferation, cellular assembly and organization, cellular function and maintenance, cell death and survival, cell morphology ranked according to –log (*P*-value) (Figure 1). Based on the activation z-score, top bio functions with a predicated increased activation state were organismal death, Hypoplasia, morphology of embryonic tissue, growth failure, while top bio functions with a decreased activation state were kidney development, quantity of gonadal cells, size of body, cell survival (Figure 2).

### Gene network analysis

**Figure 3:**
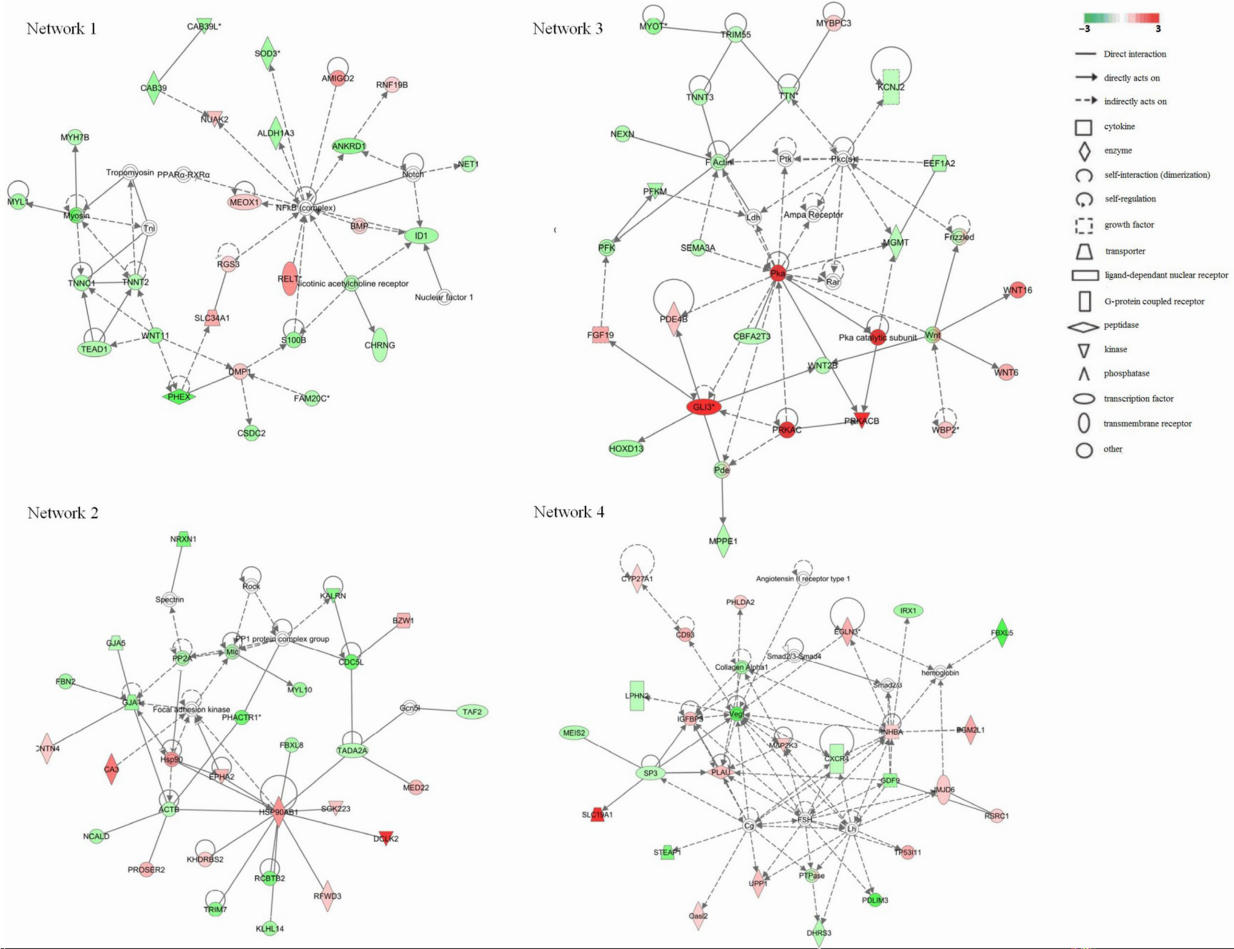
**Significant four-gene networks of 460 differentially expressed genes analyzed with the IPA program**. Molecular interactions among important focus molecules are displayed. Node (gene) and edge (gene relationship) symbols are described in the key. The intensity of the node color indicates the degree of up-(red) or down- (green) regulation; The color-change scale bar is a log_2_ scale. Symbols for each molecule are presented according to their molecular function and the type of interactions they participate in.

To further refine the analysis of the 2199 genes, we investigated their biological interactions by using the IPA program, and found 460 differential expressed genes with functional relationships were mapped to interactive gene networks. Insertion of REV *LTR* caused 25 possible gene networks in MDV-infected CEF cells (*P* ≤ 0.05). The top 10 gene networks were listed in Table 2. Gene networks with high-scoring are showed in Figure 3 and are associated with hematological disease, metabolic disease, connective tissue disorders, cell-to-cell signaling and interaction, nervous system development and function, tissue development, cardiovascular disease, embryonic development, organismal development, cellular movement, cellular function and maintenance, cellular growth, and proliferation. The remain top gene networks are involved in bio functions related to humoral immune response, protein synthesis, amino acid metabolism, ophthalmic disease, renal and urological system development and function, dermatological diseases and conditions, gastrointestinal disease, molecular transport, small molecule biochemistry, connective tissue development and function, digestive system development and function (Table 2).

**Table 2:** **The highest scoring networks (*P* ≤ 0.05)**.

**ID**	**Molecules in networks (focus genes)**	**Score**	**Number of focus genes**	**Top functions**
1	*ALDH1A3, **AMIGO2**, ANKRD1, CAB39, CAB39L, CHRNG, CSDC2, **DMP1**, FAM20C, ID1, **MEOX1**, MYH7B, MYL1, NET1, **NUAK2**, PHEX, **RELT**, **RGS3**, **RNF19B**, S100B, **SLC34A1**, SOD3, TEAD1, TNNC1, TNNT2, WNT11*	38	26	Hematological disease, metabolic disease, connective tissue disorders
2	*ACTB, **BZW1**, **CA3**, CDC5L, **CNTN4**, **DCLK2**, **EPHA2**, FBN2, FBXL8, GJA1, GJA5, **HSP90AB1**, KALRN, **KHDRBS2**, KLHL14, **MED22**, MYL10, NCALD, NRXN1, PHACTR1, **PROSER2**, RCBTB2, **RFWD3**, **SGK223**, TADA2A, TAF2, TRIM7*	34	27	Cell-to-cell signaling and interaction, nervous system development and function, tissue development
3	*CBFA2T3, EEF1A2, **FGF19**, **GLI3**, HOXD13, KCNJ2, MGMT, MPPE1, MYBPC3, MYOT, NEXN, **PDE4B**, PFKM, **PRKACB**, SEMA3A, TNNT3, TRIM55, TTN, **WBP2**, **WNT6**, **WNT16**, WNT2B*	29	22	Cardiovascular disease, embryonic development, organismal development
4	***CD93**, CXCR4, **CYP27A1**, DHRS3, **EGLN3**, FBXL5, GDF9, **IGFBP3**, **INHBA**, IRX1, **JMJD6**, LPHN2, **MAP2K3**, MEIS2, **Oasl2**, PDLIM3, **PGM2L1**, **PHLDA2**, **PLAU**, **RSRC1**, **SLC19A1**, SP3, STEAP1, **TP53I11**, **UPP1***	29	25	Cellular movement, cellular function and maintenance, cellular growth and proliferation
5	***CD83**, CEP63, CTH, **DUSP4**, EGFL6, EPB41, EXOC4, **FRAS1**, GCLM, HDGFRP3, IFNAR2, IFT88, IL13RA1, MAP6, **NDE1**, PITX1, **POLQ**, PPP1R3B, SASH3, SYNE1, TBX4*	27	21	Humoral immune response, protein synthesis, amino acid metabolism
6	*ACE, AKR1B10, CTSL2, FBLN2, FBLN5, **FGA**, FGF7, FIGF, GFRA2, **GIT1**, GREM1, MAPKAPK5, OPTC, **OSMR**, PDE5A, RASD1, RGL1, **RIT1**, **SCIN**, **TFF2**, WNT4*	26	21	Ophthalmic disease, cellular movement, renal and urological system development and function
7	*CD82, COL17A1, COL1A2, COL4A3, COL6A3, HAS2, ITGB1BP1, **MGLL**, MMP11, MMP27, MMP23B, NOV, NRG4, **PLEKHA1**, PRR5, RELN, **SEMA7A**, SNCB, STMN3, THBS2*	26	20	Connective tissue disorders, dermatological diseases and conditions, gastrointestinal disease
8	*AANAT, CLEC3B, **COX7A2**, **CYB5A**, **DACH1**, **DHCR7**, GCG, **HK2**, KCNIP1, **MERTK**, **NKX6-2**, **NTS**, PDCD4, **PRKX**, **RPS6KB1**, **SCAP**, **SH3PXD2B**, SIX6, SLC9A3R1, TUB*	26	20	Cellular function and maintenance, molecular transport, small molecule biochemistry
9	***CHIC2**, CTTNBP2, GATA2, GLI2, HDAC4, **HOXA1**, HOXD12, KCNA2, NECAB1, PARD3, PBX1, RTN1, RUNX1T1, SERPINF1, SNW1, TARS, TFAP2B, **VDR**, WT1, **ZBTB16***	25	20	Connective tissue development and function, embryonic development, organ development
10	*ACTN2, CAMK4, **CCK**, EPHX2, FBXO32, FGF18, G6PC2, HBP1, MYH1, MYOZ1, MYOZ2, PALLD, PARVG, **PPP1R16B**, PPP1R1C, PPP2R3A, PPP3CA, PRRX1, PRRX2, SRL, TMOD1*	23	21	Digestive system development and function, embryonic development, organ development

*Up-regulated genes were indicated in bold letters, the other genes were down-regulated*.

### Canonical pathway analysis

As analyzed by the IPA canonical pathway analysis program, 460 differential expressed genes were annotated to 56 metabolic and signaling pathways in the IPA canonical pathway library (Table 3, *P* ≤ 0.05). More than 20 pathways directly related to tumors were modulated in GX0101-infected CEF cells compared to GX0101Δ*LTR*-infected ones. Four pathways directly related to atherosclerosis caused by MDV infection were regulated by the insertion of REV *LTR*: atherosclerosis signaling; cysteine biosynthesis III; extrinsic prothrombin activation pathway; cysteine biosynthesis/homocysteine degradation. A series of complex immune changes that related to crosstalk between dendritic cells and natural killer cells, communication between innate and adaptive immune cells and T helper cell differentiation were also found.

**Table 3:** **List of the genes in significant canonical pathways (*P* ≤ 0.05)**.

**Ingenuity canonical pathways**	**−log(*p*-value)**	**Ratio**	**Molecules**
Axonal guidance signaling	5.60	6.4 × 10^−2^	***PRKACB**, ADAMTS7, **RGS3**, GLI2, **BMP2**, **PIK3R5**, MMP13, **WNT16**, **WNT6**, FZD1, **GLI3**, **NTRK1**, FIGF, WNT4, **ARPC3**, MYL10, PPP3CA, ACE, KALRN, CXCR4, WNT2B, **GIT1**, **PDGFB**, MYL1, SEMA3A, NTRK3, **FZD3**, **EPHA2**, WNT11, **SEMA7A***
Hepatic fibrosis/hepatic stellate cell activation	5.27	1.03 × 10^−1^	***IL8**, MYH6, **IFNGR2**, MMP13, **IL6**, IFNAR2, MYH7B, MYL1, **PDGFB**, COL1A2, **IGFBP3**, EDNRA, FIGF, MYH1, IL4*
Role of Osteoblasts, Osteoclasts, and Chondrocytes in Rheumatoid Arthritis	5.01	7.98 × 10^−2^	*CAMK4, SFRP2, SMAD9, **BMP2**, WNT2B, **PIK3R5**, **WNT16**, MMP13, **WNT6**, **IL6**, FZD1, ITGB3, BMPR1B, **FZD3**, WNT4, **MAP2K3**, WNT11, PPP3CA, IL4*
Ovarian cancer signaling	4.80	9.86 × 10^−2^	***PRKACB**, **RPS6KB1**, GJA1, **FGF9**, WNT2B, **PIK3R5**, **WNT16**, **WNT6**, FZD1, **FZD3**, EDNRA, WNT4, FIGF, WNT11*
Basal cell carcinoma signaling	4.71	1.37 × 10^−1^	*GLI2, **GLI3**, **BMP2**, WNT2B, **FZD3**, **WNT16**, WNT4, **WNT6**, FZD1, WNT11*
Human embryonic stem cell pluripotency	4.65	8.97 × 10^−2^	***BMP2**, WNT2B, **PIK3R5**, **WNT16**, **WNT6**, FZD1, **PDGFB**, **INHBA**, BMPR1B, NTRK3, **NTRK1**, **FZD3**, WNT4, WNT11*
Bladder cancer signaling	4.62	1.21 × 10^−1^	*MMP27, **IL8**, MMP23B, FGF18, **FGF9**, MMP13, FIGF, MMP11, FGF7, FGF13, **FGF19***
Actin cytoskeleton signaling	4.59	7.56 × 10^−2^	*MYH6, FGD3, **FGF9**, ACTB, ACTN2, **PIK3R5**, MYH7B, **GIT1**, TTN, MYL1, **PDGFB**, FGF13, FGF18, **ARPC3**, MYL10, FGF7, **FGF19**, MYH1*
Regulation of the epithelial-mesenchymal transition pathway	4.51	8.38 × 10^−2^	*TWIST2, **FGF9**, WNT2B, **PIK3R5**, **WNT16**, **WNT6**, FZD1, FGF13, PYGO1, FGF18, **FZD3**, WNT4, **MAP2K3**, FGF7, WNT11, **FGF19***
Role of NANOG in mammalian embryonic stem cell pluripotency	3.76	9.65 × 10^−2^	*BMPR1B, SMAD9, **BMP2**, WNT2B, **FZD3**, **PIK3R5**, **WNT16**, WNT4, **WNT6**, FZD1, WNT11*
Role of macrophages, fibroblasts, and endothelial cells in rheumatoid arthritis	3.57	6.02 × 10^−2^	***IL8**, CAMK4, SFRP2, IL15, WNT2B, **PIK3R5**, **WNT16**, MMP13, **WNT6**, **IL6**, FZD1, **PDGFB**, **IL16**, TLR7, **FZD3**, WNT4, FIGF, **MAP2K3**, WNT11, PPP3CA*
Clathrin-mediated endocytosis signaling	3.25	7.18 × 10^−2^	*APOA1, **FGF9**, ACTB, **PIK3R5**, **PDGFB**, FGF13, ITGB3, ALB, FGF18, **ARPC3**, FIGF, FGF7, PPP3CA, **FGF19***
Colorectal cancer metastasis signaling	3.02	6.2 × 10^−2^	***PRKACB**, WNT2B, **PIK3R5**, **WNT16**, MMP13, **WNT6**, **IL6**, FZD1, MMP27, MMP23B, **FZD3**, TLR7, WNT4, FIGF, MMP11, WNT11*
Agranulocyte adhesion and diapedesis	2.87	6.99 × 10^−2^	*MMP27, **IL8**, MYH6, MMP23B, CXCR4, ACTB, CXCL14, MMP13, **CCL20**, MMP11, MYH7B, MYL1, MYH1*
Cellular effects of sildenafil (Viagra)	2.84	7.48 × 10^−2^	***PRKACB**, MYH6, CACNG1, CAMK4, ACTB, PDE5A, **PDE4B**, MYL10, MYH7B, MYL1, MYH1*
Calcium signaling	2.76	6.13 × 10^−2^	***PRKACB**, CHRNG, MYH6, HDAC4, CAMK4, TNNT3, TNNC1, TNNT2, MYH7B, PPP3CA, CASQ2, MYL1, MYH1*
Retinoate biosynthesis I	2.58	1.35 × 10^−1^	*AKR1B10, ALDH1A3, **SDR16C5**, **BMP2**, ADH1C*
Crosstalk between dendritic cells and natural killer cells	2.53	8.42 × 10^−2^	*ACTB, IL15, TLR7, TNFSF10, **CD83**, **IL6**, **HLA-DRB5**, IL4*
Role of tissue factor in cancer	2.48	7.83 × 10^−2^	***IL8**, **RPS6KB1**, **PIK3R5**, MMP13, **HBEGF**, **F7**, **FGA**, STAT5B, ITGB3*
Inhibition of matrix metalloproteases	2.37	1.25 × 10^−1^	*MMP27, MMP23B, THBS2, MMP13, MMP11*
Role of Wnt/GSK-3β signaling in the pathogenesis of influenza	2.30	8.64 × 10^−2^	*WNT2B, **FZD3**, **WNT16**, WNT4, **WNT6**, FZD1, WNT11*
Atherosclerosis signaling	2.26	6.62 × 10^−2^	*COL1A2, **IL8**, ALB, CXCR4, APOA1, **PLA2G10**, MMP13, **IL6**, **PDGFB***
TGF-β signaling	2.15	7.87 × 10^−2^	*BMPR1B, **AMH**, SMAD9, **BMP2**, **MAP2K3**, **VDR**, **INHBA***
Superpathway of methionine degradation	2.13	9.38 × 10^−2^	*CBS, MAT1A, MGMT, MCEE, CTH, CDO1*
Glioblastoma multiforme signaling	2.10	6.1 × 10^−2^	***RPS6KB1**, WNT2B, **FZD3**, **PIK3R5**, **WNT16**, WNT4, **WNT6**, FZD1, **PDGFB**, WNT11*
Cysteine biosynthesis III (mammalia)	2.09	1.33 × 10^−1^	*CBS, MAT1A, MGMT, CTH*
Extrinsic prothrombin activation pathway	2.00	1.5 × 10^−1^	***F7**, **TFPI**, **FGA***
FGF signaling	1.99	7.69 × 10^−2^	*FGF18, **FGF9**, **PIK3R5**, **MAP2K3**, FGF7, FGF13, **FGF19***
Communication between innate and adaptive immune cells	1.94	6.42 × 10^−2^	***IL8**, IL15, TLR7, **CD83**, **IL6**, **HLA-DRB5**, IL4*
Oncostatin M signaling	1.89	1.14 × 10^−1^	*MMP13, **OSMR**, **PLAU**, STAT5B*
ILK signaling	1.89	5.73 × 10^−2^	*MYH6, PPP2R3A, ACTN2, ACTB, **BMP2**, **PIK3R5**, FIGF, MYH7B, MYL1, MYH1, ITGB3*
Coagulation system	1.85	1.05 × 10^−1^	***F7**, **PLAU**, **TFPI**, **FGA***
IL-4 signaling	1.78	7.59 × 10^−2^	***RPS6KB1**, IL13RA1, **INPP5F**, **PIK3R5**, **HLA-DRB5**, IL4*
Role of IL-17A in arthritis	1.76	7.94 × 10^−2^	***IL8**, **PIK3R5**, **CCL20**, MMP13, **MAP2K3***
Wnt/β-catenin signaling	1.75	5.75 × 10^−2^	*GJA1, SFRP2, PPP2R3A, WNT2B, **FZD3**, **WNT16**, WNT4, **WNT6**, FZD1, WNT11*
Cysteine biosynthesis/homocysteine degradation	1.74	2.5 × 10^−1^	*CBS, CTH*
HIF1α signaling	1.65	6.54 × 10^−2^	*MMP27, MMP23B, **PIK3R5**, MMP13, FIGF, **EGLN3**, MMP11*
TREM1 Signaling	1.64	7.04 × 10^−2^	***IL8**, TLR7, **CD83**, **IL6**, STAT5B*
PPARα/RXRα activation	1.63	5.24 × 10^−2^	***PRKACB**, GHR, **HSP90AB1**, APOA1, FASN, **MAP2K3**, **IL6**, STAT5B, **TGS1**, ABCA1*
RAR activation	1.63	5.29 × 10^−2^	***PRKACB**, SMAD9, ALDH1A3, **SDR16C5**, **BMP2**, ADH1C, **IGFBP3**, SNW1, STAT5B, **ZBTB16***
Role of JAK family kinases in IL-6-type cytokine signaling	1.54	1.11 × 10^−1^	***OSMR**, **IL6**, STAT5B*
Factors promoting cardiogenesis in vertebrates	1.52	6.38 × 10^−2^	*BMPR1B, SMAD9, **BMP2**, **FZD3**, FZD1, WNT11*
Nitric oxide signaling in the cardiovascular system	1.50	6.06 × 10^−2^	***PRKACB**, CAMK4, **HSP90AB1**, **CAV1**, **PIK3R5**, FIGF*
Neuroprotective role of THOP1 in Alzheimer's disease	1.46	7.41 × 10^−2^	***PRKACB**, **NTS**, TAC1, ACE*
IL-15 production	1.45	9.68 × 10^−2^	*IL15, **IL6**, **IRF1***
Hematopoiesis from multipotent stem cells	1.40	1.67 × 10^−1^	*IL15, IL4*
Integrin signaling	1.39	4.83 × 10^−2^	*ACTN2, ACTB, ITGA8, **CAV1**, **PIK3R5**, **ARPC3**, TSPAN6, **GIT1**, TTN, ITGB3*
T helper cell differentiation	1.39	6.94 × 10^−2^	***IFNGR2**, **IL6**, GATA3, **HLA-DRB5**, IL4*
PTEN signaling	1.37	5.26 × 10^−2^	***RPS6KB1**, BMPR1B, GHR, **INPP5F**, NTRK3, **NTRK1**, **PIK3R5***
Granulocyte adhesion and diapedesis	1.36	5.14 × 10^−2^	*MMP27, **IL8**, MMP23B, CXCR4, CXCL14, **CCL20**, MMP13, MMP11, ITGB3*
AMPK signaling	1.34	4.79 × 10^−2^	***PRKACB**, CAB39, **RPS6KB1**, PPP2R3A, FASN, **PIK3R5**, **MAP2K3**, PFKM*
Epithelial adherens junction signaling	1.34	5.44 × 10^−2^	*MYH6, ACTN2, ACTB, **ARPC3**, PARD3, MYH7B, MYL1, MYH1*
Sonic hedgehog signaling	1.34	9.09 × 10^−2^	***PRKACB**, GLI2, **GLI3***
Role of IL-17A in psoriasis	1.33	1.54 × 10^−1^	***IL8**, **CCL20***
Growth hormone signaling	1.33	6.58 × 10^−2^	***RPS6KB1**, GHR, **IGFBP3**, **PIK3R5**, STAT5B*
BMP signaling pathway	1.33	6.25 × 10^−2^	***PRKACB**, BMPR1B, CAMK4, SMAD9, **BMP2***

*Up-regulated genes were indicated in bold letters, the other genes were down-regulated*.

## Discussion

The insertion of REV *LTR* into MDV genome is associated with alteration of biological properties of MDVs, and may further provides a selective force for virus evolution (Isfort et al., 1994; Jones et al., 1996; Witter et al., 1997). Herein, we studied the host response changes to MDV infection by an integrated *LTR in vitro* using high-throughout microarray analysis. We found that some differentially expressed genes that have already been reported as such for the MD system were also significantly altered in our study (reviewed by Haq et al., 2010), but majority differentially expressed genes were reported for the first time.

The insertion of REV *LTR* changed the expressed transcription profiles of host cells after MDV infection that associated with cell-to-cell signaling and interaction appear to be a common strategy for the host responses to stress caused by virus infection. The oxidants generated in MD causes significant damage to DNA (Keles et al., 2010). In response to stress, *HSP90* promotes the maturation, structural maintenance and proper regulation of specific target proteins involved for instance in cell cycle control and signal transduction as a molecular chaperone (Chen et al., 2015). While *Hsp90* is an vital protein that mediates the infectivity and replication of some herpes-viruses, such as herpes simplex virus type 1 (Burch and Weller, 2005), Epstein-Barr virus (Sun X. et al., 2010) and Kaposi's sarcoma-associated herpesvirus (Wen and Damania, 2010), the transcription level is reduced in MDV-infected chickens (Hu et al., 2012). The inserted REV *LTR* activated transcription of *HSP90* and might play a negative role in regulating cell cycles after MDV infection. A set of proteins that interact with *HSP90* and involve in promoter responses to many activators and repressors were also modulated. *CDC5L*, DNA binding and poly(A) RNA binding related proteins, that take part in signal transduction involved in DNA damage checkpoint, were down-regulated; on the contrary, *RFWD3* has a positive regulation role in defense response to virus by host regulation of DNA damage checkpoint, was up-regulated (Gong and Chen, 2011). Thus, the insertion of REV *LTR* regulated host cell cycles and DNA damage checkpoint in response to stress by MDV infection, both of which were closely associated with tumor formation. In fact, we found that more than 20 pathways directly related to tumors were modulated in GX0101-infected CEF cells compared to GX0101Δ*LTR*-infected CEF cells. From various cancer formation and metastasis signals, it can be seen that a set of matrix metalloproteases (MMPs) (*MMP27, MMP23B, THBS2, MMP13, MMP11*) as well as the MMPs receptor *ITGB3* were inhibited in GX0101-infected CEFs. MMPs are members of an enzyme family that are critical for maintaining tissue allostasis and are required for tumor invasion and metastasis by destroy extracellular matrix (Clark et al., 2008). In line with this, oncostatin M signaling that inhibits the proliferation of some tumor cells (Krona et al., 2007) was activated. RAR signal that plays a role in anti-cancer aspect by suppress the proliferation of tumor cells and promote apoptosis was also activated (Brigger et al., 2015). Therefore, there is a lower chances for GX0101 with a REV *LTR* to induce tumors *in vivo*. This provides some clues and support the report that the insertion of REV *LTR* into MDV genome is closely related to the tumor formation *in vivo* (Jones et al., 1996; Witter et al., 1997). The REV *LTR* shows strong promoter or enhancer activity, and various genes are transactivated depending on the location of the insertion (Jones et al., 1993, 1996). The insertion of REV *LTR* into RM1 attenuate the oncogenicity of the virus *in vivo* replication (Witter et al., 1997). The field MDV strain GX0101 with a REV *LTR*, shows a lower but not significant oncogenicity *in vivo* (Sun A. J. et al., 2010). Our study have provided some evidence that the GX0101 with a REV *LTR* indeed showed a lower oncogenicity from the aspect of host gene expression profiles.

The growth inhibition and early mortality syndrome caused by MDV infection might be altered by the inserted REV *LTR* in MDV genome. Our results showed that REV *LTR* caused predicated increased activation state of organismal death and growth failure, while decreased activation state of size of body, cell survival. In line with this, Network 3 clustered a set of genes which had top functions associated embryonic development and organismal development. WNT signal was activated as indicated by the up-regulation of *WBP2, WNT6*, and *WNT16* genes. WNT signal integrates signal (like *retinoic acid, FGF, TGF*-β, and *BMP*) delivered by other pathways and regulates the embryonic and organismal development. In addition, protein kinase A (Pka) signal pathway was supposed to be significantly up-regulated in the current gene expression mode. Pka system is one of the G protein-coupled system signal transduction pathways. *GPCRs* is activated by various external stimuli, which further activates the downstream MAPK cascade signaling as revealed by network 4. Activation of MAPK cascade is an essential event in various cellular activity including cellular growth and proliferation, and cellular movement and death, those are the top functions of network 4. In our study, we found that *LTR* up-regulated key activators in the pathway like *IGFBP3, INHBA*, and *MAP2K3*, which initiate downstream MAPK cascade signaling. *IGFBP3* can positive regulate the insulin-like growth factor receptor and growth factor; *INHBA* takes part in activating the Erk5/BMK1 MAPK cascade signal leading to cell growth, differentiation and development; *MAP2K3* can activate *TAK1* that mediates p38 MAPK activation and plays a role in inflammation, apoptosis, growth and differentiation. Other differential expressed genes identified (Table 2) that associated to the cell differentiation and proliferation, and cellular function and maintenance were also up-regulated, for example, *EGLN3*, that responses to hypoxia and regulate cell proliferation; *PHLDA2*, that plays a key role in organ morphogenesis, regulation of gene expression, cell migration, and embryonic development; *TP53I11*, that responses to stress and regulate cell transcriptional activity and apoptosis. The combined results from the above studies indicated that the insertion of REV *LTR* into MDV GX0101 genome had a greater growth and survival inhibition on chickens caused by GX0101 infection. Previous *in vivo* study demonstrated that GX0101 with a REV *LTR* insert in the genome was attenuated for pathogenic effects with lower growth and mortality rates of the infected birds during 100 days post infection (Sun A. J. et al., 2010). The *in vivo* and *in vitro* experiments seem contradictory and this is probably because that the MDV infection cellular model may not represent the whole infection phase, while indicates the productive infection phase of MDV infection *in vivo*.

It has long been recognized that atherosclerosis and nervous system disorders are clinically distinct disease syndromes caused by MDV infection (Fabricant and Fabricant, 1999). Four pathways directly related to atherosclerosis caused by MDV infection were regulated by the insertion of REV *LTR*. *PLA2G10*, an indicator of atherosclerosis inflammatory (Piñón and Kaski, 2006), was significantly up-regulated in GX0101-infected cells. On the contrary, most of the genes in these pathways were down-regulated, such as *CBS, CTH, F7, MGMT, MAT1A, COL1A2, ALB, CXCR4, APOA1, MMP13*. It was reported that *MAT1A* and *APOA1* were required to maintain normal cardiovascular system (Andrikoula and McDowell, 2008). Myosins, that plays a vital role in muscle contraction and involves in a wide range of other motility activities in eukaryotes, were predicted to be down-regulated by a set of calcium ion binding proteins and associated proteins including *MYL1, TNNC1, TNNT2, MYH7B, TEAD1*, and *WNT11* (Leslie, 2015). A group of genes involved in F-actin protein were highly down-regulated (*NEXN, TNNT3, TRIM55, TTN, MYOT*). Actin family proteins have a close relationship with atherosclerosis pathogenesis (Berceli et al., 1991). Therefore, The insertion of REV *LTR* can alter the atherosclerosis status caused by MDV infection. Besides, four genes were clustered to play a protective role in neurodegenerative diseases, including two up-regulated genes, *PRKACB* and *NTS*, and two down-regulated genes, *TAC1* and *ACE*. *ID1, CHRNG*, and *S100B* were the three genes that directly related to a predicted down-regulated nicotinic acetylcholine receptor that play important roles in the peripheral nervous system (Pillai et al., 2011). These genes might related to the modified nervous system disorders in MDV-infected chickens caused by the insertion of REV *LTR* into MDV genome; the functions of all remain to be investigated in the future.

The insertion of REV *LTR* is also associated with a series of complex immune changes that related to crosstalk between dendritic cells and natural killer cells, communication between innate and adaptive immune cells and T helper cell differentiation in MDV infections. The gene array showed increased transcription of *IL-6, IL-8, IRF1, CD83, HLA-DRB5, IFNGR2, CCL20* while decreased transcription of *IL-4, IL-15, TLR7, TNFSF10, IL13RA1*. *IL-6* is involved in the acute phase immune response and involved in lymphocyte and monocyte differentiation (Mayer et al., 1991). It plays an essential role when B-cells are finally differentiated into Ig-secreting cells. *IL-4* induces the expression of MHC class II molecules on resting B-cells and activates the process of several immune cell activation including B-cells (Ryzhov et al., 2004). *CD83* may be involved in the antigen presentation or the cellular interactions after the activation of lymphocyte and negative regulates *IL-4* production (Roy et al., 2004). *IL13RA1* is an alternate accessory protein to the gamma chain of the common cytokine receptor in IL-4 signal transduction pathway (Miloux et al., 1997). Taken together, the insertion of REV *LTR* into MDV genome might stimulate the differentiation of B-cells to Ig-secreting cells, while inhibited the B-cell activation process. Therefore, the host humoral immunity was dysregulated by the insertion of REV *LTR* into MDV genome, even though that no obvious changes of antibody titer was observed *in vivo* study (Sun A. J. et al., 2010). *IL-15* that stimulates the proliferation of T-lymphocytes and mediate the crosstalk between dendritic cells and natural killer cells was down-regulated (Boudreau et al., 2011). *TRL7* immediate innate immune response that plays a role in defense response to virus was also down regulated. This indicated that the REV *LTR* may help GX0101 to introduce a immune evasion mechanism to escape the host innate and adaptive immune responses. *IL-8* is secreted by several immune cells in response to an inflammatory stimulus and serves as a chemotactic factor that attracts a set of cell types except monocytes. Neutrophils can be activated by *IL-8*. *CCL20* is hemotactic factor that mainly attracts lymphocytes. The increased transcription of *IL-8* and *CCL20* indicated a up-regulated inflammatory status in GX0101-infected cells compared to GX0101Δ*LTR*. In addition, retinoate biosynthesis I was also influenced in MDV-infected cells by the insertion of REV *LTR* with the activation of retinoic acids receptors (RAR). Retinoic acids (RA) participates in apoptosis process of T lymphocytes through the interaction of RARs and RXRs (Szondy et al., 1998) and mediates the Th1 to Th2 polarization via RARs (Iwata et al., 2003). RA is closely related to thymocytes apoptosis. Thus, the insertion of REV *LTR* might relate to thymus atrophy in MDV infections by influencing of the RA signal. In addition, the microarray analysis of host cells in responses to different MDVs infection also detected a various of other pathways that take part in virus infection. For instance, clathrin-mediated endocytosis signaling that mediated virus endocytosis by the inward budding of plasma membrane vesicles was modified (Reis et al., 2015). Wnt/GSK-3β signaling that plays a role in the pathogenesis of influenza was also modulated (Hiyoshi et al., 2015). The precise role of the indicated pathways in the differential host responses after the insertion of REV *LTR* into MDV genome have not been verified, further investigations are being performed to identify unique and more deeply involved interactions between host cells and different MDVs.

Taken together, our study using microarray analysis of CEF cells infected with MDVs with/without REV *LTR*, provides novel insights on the mechanistic basis of how REV *LTR* alters the pathogenesis of the productive infection phase of MDV infection *in vitro*. In the study, a number of differential transcript genes were clustered to a large number of biological pathways according to their biological functions. The insertion of REV *LTR* showed the greatest influence on cancer formation and metastasis, followed with immune changes, atherosclerosis and nervous system disorders. Based on the bio functions, GX0101 infection was predicated with a greater growth and survival inhibition but lower oncogenicity on chickens than GX0101Δ*LTR*, at least in the acute phase of infection. More detailed investigation should be carried out to eliminate the possible relationship between these differential genes with the selective advantages for GX0101 to become the predominant strain that could be isolated at higher frequency.

## Author contributions

NC and HH collection and assembly of the data, manuscript writing, and data analysis; XL, NC, and SS discussion, manuscript revision; SS and ZC concept and design, data analysis, manuscript revision, and final approval of the manuscript.

### Conflict of interest statement

The authors declare that the research was conducted in the absence of any commercial or financial relationships that could be construed as a potential conflict of interest.

## Abbreviations

MD: Marek's disease
MDV: Marek's disease virus
REV: reticuloendotheliosis virus
*LTR*: long terminal repeat
CEF: chicken embryo fibroblasts
DEF: duck embryo fibroblasts
*GAPDH*: Glyceraldehyde-3-phosphate dehydrogenase.
