# Supplementary material for: Transcriptional and Bioinformatic Analysis Provide a Relationship between Host Response Changes to Marek's Disease Viruses Infection and an Integrated Long Terminal Repeat
Source: Front Cell Infect Microbiol. 2016 Apr 26;6:46. doi: 10.3389/fcimb.2016.00046 (PMC4844599; doi:10.3389/fcimb.2016.00046)
Supplement: Supplementary file 1 [file Table1.PDF]

GeneName	fGvsL	log2f
TC223676	189.5458722	7.566403229
ENSGALT0000<br>0002900.2	165.1664194	7.367776586
BU236185.1	62.75684903	5.971701013
CR385114	53.98585606	5.754509575
AY957508	36.8823391	5.20485825
CV891990.1	30.70837631	4.940560327
CR390383	27.79061295	4.79652575
GLI3	27.63512122	4.788431036
SLC19A1	26.3203784	4.718108325
CR385943.1	25.11655533	4.650566711
BU109828	21.08568163	4.398191755
AJ851527	20.71710808	4.372750725
CR390471	19.00213531	4.248089641
CR387852	18.06581077	4.175190097
BU111473	17.29238432	4.1120649
CN230868	16.85604178	4.07519389
CR387135	16.28571341	4.025535014
AJ719619	15.4522332	3.94974345
BM439709	15.29047938	3.934561733
CR405842	14.95958314	3.902998069
CR386526	14.84619893	3.8920217
BU481434	14.22944502	3.83080749
BU304307	13.94865308	3.802053913
BU450117	13.64226032	3.770010793
TC207215	12.6426888	3.660231418
BU461111	12.60600448	3.656039175
CR352972	12.13260468	3.600817402
CR391652	11.12787409	3.476106096
BX931541	10.64241933	3.41175425
AL584430	10.5760287	3.402726092
BU270589	10.56483569	3.401198425
TC188468	10.53291828	3.396833304
TC203237	10.31310875	3.366407375
SH3PXD2B	10.19093298	3.349214231
BU106680	10.11142412	3.3379143
BU201531	10.08761041	3.334512559
BU476939	9.989822914	3.320459104
BU454442	9.840260366	3.298696489
FRAS1	9.439751328	3.238748855

CR524348	9.433352851	3.237770632
MGLL	9.313518673	3.219326325
BX258989	9.034937211	3.175514575
BU456885	8.887540268	3.151784191
WNT16	8.788451471	3.135608984
CR352666	8.692518838	3.119774288
AJ743462	8.519597021	3.090785192
CN227279	8.327554349	3.057892865
TC223891	8.028904737	3.005203196
CN237811	7.962176718	2.993162892
BU399778	7.890568515	2.98012925
BU296667	7.837697521	2.970429896
CR523741	7.826794682	2.9684216
CR390278	7.60502751	2.926953467
CR385987	7.511044542	2.909013554
RCJMB04_16f17	7.319620023	2.871768757
TC204457	7.250999479	2.85817987
TC195480	7.21011308	2.850021886
BU255000	7.201717458	2.848341
TC214683	7.157340748	2.839423665
BX930588	7.023981721	2.81228909
TC225121	7.006721793	2.808739614
L16955	7.002755661	2.80792275
AM070792	6.680741549	2.740008248
CN233134	6.627287469	2.7284185
BX932557	6.554123399	2.712402836
X61193	6.512864219	2.70329215
CR386058	6.503168243	2.701142748
CN230951	6.482576207	2.696567261
BU289261	6.430053298	2.684830696
DR413254.1	6.412955979	2.680989504
BU468651	6.363049854	2.669718425
CR407478	6.004189898	2.585969607
CR391440	5.948115357	2.572432627
HOXA1	5.934045354	2.569015954
Y14971	5.9210346	2.565849284
AY849319	5.915626175	2.564530887
TC201114	5.883359434	2.556640178
CN227033	5.782652017	2.531731288
ZBTB16	5.703442	2.511832842

TC218598	5.696077691	2.509968824
BX950703	5.66140375	2.501159815
CR405945	5.589925961	2.482829175
CN237026	5.518951329	2.464394163
BX931022	5.493483666	2.457721318
X70101	5.493209499	2.457649314
BX935630	5.383830635	2.428633026
BU276425	5.35346108	2.420471914
BX932821	5.340334733	2.416930173
CK608429	5.280464335	2.400664798
CN229773	5.261533722	2.395483402
CR386390	5.239384824	2.38939743
TC210527	5.209350146	2.381103411
BU201302	5.203452266	2.379469107
AF189767	5.159663916	2.367277096
TC223191	5.137157152	2.360970208
TC203813	5.088011183	2.347101842
AF066861	5.046377036	2.335248
L34552	5.014839764	2.326203604
CR733120	5.001580378	2.322384023
TC202639	4.99648099	2.320912366
AJ455565	4.971501337	2.313681595
CR387097	4.966268371	2.312162225
BX932486	4.961000966	2.310631238
BX931604	4.942764243	2.305318095
CN229862	4.940000882	2.304511299
BU308744	4.909079291	2.295452469
CN229711	4.902671202	2.29356801
TC226494	4.827368965	2.271237099
TC227280	4.799238466	2.2628055
AF022151	4.78257774	2.257788419
CR524374.1	4.758943999	2.250641478
BU236926	4.757411706	2.250176882
TC225199	4.728274999	2.241313946
CO772113.1	4.69379242	2.230754041
BU257807	4.690968115	2.229885695
BU444229	4.686147598	2.228402395
CR732803	4.624133577	2.209183074
CR388809	4.597747645	2.200927284
AJ454418.1	4.535230114	2.181175754
BU432506	4.496627106	2.16884325

CR354122	4.417663648	2.143283579
CR389854	4.385687062	2.132802873
AL587676.1	4.32140632	2.111500887
CR354088	4.310659831	2.107908719
AJ851707	4.304584104	2.105873855
BU421706	4.291263389	2.101402454
TC223371	4.254056008	2.088839028
BU450824	4.253904529	2.088787655
CR391187	4.252658775	2.088365101
TC195661	4.245965517	2.086092655
BE140008	4.24348295	2.085248881
CR390698	4.21485193	2.075481949
CR354003	4.210817455	2.074100334
BU418601	4.207624478	2.073005953
BU216001	4.159143655	2.056286516
BX934166	4.15218895	2.053872097
BU213122	4.117421634	2.041741192
BX929762	4.106829324	2.038024992
TC217652	4.028287258	2.010166566
TC223240	4.022149453	2.00796669
BX932738	3.94645837	1.980558531
BU421898	3.926502228	1.973244716
TC225408	3.920791436	1.971144901
CN225251	3.916653874	1.969621639
AF315355	3.904574599	1.965165376
BX950412	3.890827699	1.960077094
PGM2L1	3.869441321	1.952125282
BM486913	3.867439441	1.951378701
BU415266	3.850733175	1.945133159
CR385843	3.846891194	1.943693025
TC223069	3.846822452	1.943667244
AB101005	3.839218311	1.940812599
AJ719692	3.834570599	1.939065032
BX262998	3.801364905	1.926517521
AF131224	3.780392537	1.918536044
CR522946	3.762049772	1.911518936
ENSGALT00000001790.2	3.755632167	1.90905577
BX931540	3.750523378	1.907091935
TC215684	3.73730622	1.901998777
AF378331	3.730038721	1.899190607

CR352623	3.7260797	1.897658534
BU331736	3.718181639	1.89459725
CR386046	3.716551453	1.893964581
BX930150	3.704797406	1.889394652
AJ721001	3.704144716	1.889140464
CR385165	3.702250364	1.88840246
BX932836	3.689839997	1.883558258
AJ724747	3.668570779	1.87521812
BU203832	3.627032372	1.858789622
BM487153	3.622320034	1.856914015
CR389394	3.606144427	1.850457178
BX950833	3.591984125	1.844780974
EGLN3	3.581665701	1.840630688
BU355311	3.572814506	1.837061014
BX265679	3.56181941	1.832614371
LOC378902	3.5589716	1.83146042
BX932917.2	3.551939832	1.828607143
BX931037	3.547680033	1.826875898
AF011356	3.546268204	1.826301651
CR353286	3.520130751	1.815629017
TC224683	3.515458141	1.813712716
BU391244	3.502258818	1.808285704
BU215674	3.486958573	1.801969224
CR386824	3.484078584	1.800777165
CR387609	3.480429814	1.799265482
CAV1	3.472282241	1.79588422
CR385510	3.463732792	1.792327638
CR524092	3.447217248	1.785432222
BU254221	3.439279348	1.7821063
CF254450	3.439219197	1.782081068
BU247224	3.437543705	1.781378056
TC203989	3.417589216	1.772979
BU332138	3.415108584	1.77193145
CF250203	3.405298719	1.76778136
TC192578	3.387250884	1.760114847
BU448531	3.38171333	1.757754367
BU265401	3.369606716	1.752580217
CR385780	3.368073983	1.751923829
AL587278	3.366138783	1.751094659
BX950769	3.337155763	1.738619027
TC222550	3.329473565	1.735294086

BX931154	3.318850727	1.730683742
BU111856	3.310820754	1.727188906
BX264133	3.305039861	1.724667672
TC218766	3.304441631	1.724406513
CN232820	3.302392845	1.72351175
BX936089	3.292028877	1.718976991
CR353930	3.285603935	1.71615858
BU259775	3.271448853	1.709929715
BU213202.1	3.264961723	1.707066078
BX264211	3.264104272	1.706687145
CR391093	3.256839701	1.703472713
BU271568	3.248214799	1.699647038
BX930900	3.245113811	1.698269076
TC217999	3.227261211	1.690310353
CR386421	3.224389994	1.68902625
CR353110	3.217801591	1.686075372
AI982017	3.211806549	1.683385
BX272932	3.207266961	1.681344443
BX934285	3.178612738	1.668397258
BU390517	3.171204317	1.665030832
CF253440	3.160548211	1.660174822
BX934779	3.15372694	1.657057752
BU269522	3.152178621	1.656349289
TC203684	3.147389569	1.65415576
X97588	3.139497035	1.65053345
BX950574	3.135883058	1.64887176
EPHA2	3.135562561	1.648724305
AJ719647	3.128087629	1.645280928
BX934904	3.125452135	1.644064909
BX935001	3.120790978	1.641911733
BU407954	3.101239891	1.632845127
CR388777	3.091636887	1.628370885
TC186333	3.087876197	1.626614911
CR406387	3.086531414	1.625986475
CN232571	3.084131459	1.62486426
UBE2J1	3.073008603	1.619651806
AJ720675	3.065726608	1.616229048
CD731305	3.062732322	1.614819283
BM491266	3.051622947	1.609576716
LOC404534	3.038004939	1.603124215
BX931913	3.019602791	1.594358785

BU456225	3.015784457	1.59253332
CV854388	3.013830044	1.591598063
BX929354	3.009996187	1.589761659
TC205809	2.98977445	1.580036651
TC223022	2.987587382	1.57898091
CV858855	2.984030049	1.577262064
CR405865	2.981662811	1.576117116
BU236104.1	2.978256854	1.574468182
CN231021	2.969966853	1.57044683
TC221734	2.959768417	1.565484298
BU448805.1	2.955753945	1.563526176
CN235535	2.944957374	1.558246753
BU301243	2.937673886	1.55467425
TC219130	2.933752162	1.552747
AJ720090	2.931681326	1.551728291
CR389547	2.928284333	1.550055645
BU427040	2.924186008	1.548035084
TC226196	2.922360026	1.547133925
CN222581	2.917344928	1.544655971
BX930333	2.91511301	1.543551813
BU314955	2.905734662	1.538902969
U09350	2.904349254	1.538214951
BX934401	2.902831898	1.537461029
CR523011	2.898196923	1.535155625
BU120985	2.895920451	1.534021973
CR406014	2.895546971	1.5338359
BX931237	2.895387191	1.533756288
CR390169	2.89376623	1.53294838
CR390664	2.892111288	1.532123068
CR524097	2.879680427	1.525908717
TC190292	2.878493344	1.525313876
TC217058	2.874423394	1.523272582
CD735320	2.87271972	1.522417242
AJ443810	2.868186412	1.520138792
BX930338	2.868177783	1.520134452
CO423291	2.863572125	1.517815941
BU434809	2.857686228	1.514847518
TC226255	2.856129919	1.514061606
BU448200	2.84878868	1.510348608
BU325744	2.841000503	1.506399087
BU219473	2.832818774	1.50223831

IGFBP3	2.824506933	1.497999042
CR406950	2.820286062	1.495841503
DR430465	2.817535865	1.494433975
J00851	2.815551851	1.49341772
AJ309540	2.810734603	1.490947237
CR524236	2.808155203	1.489622674
AB154518	2.807094353	1.489077557
BX931625	2.805824678	1.488424865
TC198303	2.803298746	1.4871255
CR390832	2.801251275	1.486071401
CR391713	2.801199475	1.486044722
BU439737	2.795679432	1.483198943
BU123182	2.79281306	1.481719008
CO757014	2.7863788	1.478391402
BU311604	2.784613089	1.477476885
CR407347	2.782279811	1.476267518
BX932219.1	2.772765975	1.471325856
TC202477	2.760143029	1.464743029
CR523422	2.758787462	1.464034315
ISG12-2	2.75411438	1.461588477
TC227529	2.751670233	1.460307584
BU247588	2.747784253	1.458268733
CR390585	2.746716478	1.457708
BX934897	2.746044963	1.457355248
BX934885	2.745265342	1.456945599
J05187	2.74505492	1.456835013
BU290878	2.737392905	1.452802523
BU282155	2.734937757	1.451508
BX279348	2.734755422	1.451411814
BU199440	2.73370713	1.450858691
BX935969	2.733173372	1.450576976
BX258135	2.729200166	1.448478209
ENSGALT00000016539.2	2.726942082	1.447284059
BU229910	2.72648288	1.447041097
CO771740.1	2.720180244	1.44370225
BU426432	2.71376103	1.440293684
AJ719362	2.713221828	1.440007004
AF162861	2.711155986	1.438908121
BX933168	2.697341038	1.43153794
DR974323	2.695602885	1.430607975

TC208888	2.694603063	1.430072768
U76665	2.693642707	1.4295585
AJ721135	2.681644045	1.42311775
DR419836.1	2.676784317	1.420500898
CD218974	2.673141368	1.418536136
CN383541.1	2.671238254	1.417508659
BU110710	2.670765042	1.417253062
TC221485	2.669103037	1.416355
RCJMB04_39d10	2.660111065	1.411486482
BX929501	2.659517096	1.411164311
TC215227	2.65882997	1.410791521
AJ720373	2.658103232	1.410397135
CR405864	2.657808453	1.410237134
BX931327	2.656124921	1.409323
NUAK2	2.654531046	1.408457015
DHRS7	2.650004615	1.405994872
CN228414	2.644529752	1.403011206
AJ720018	2.643128314	1.402246464
CN209533	2.641189582	1.401187861
U22014	2.640697102	1.400918828
CK607032	2.634924723	1.397761746
CR387252	2.633870925	1.397184647
AJ444673	2.633549838	1.397008762
LEPREL1	2.630611511	1.395398207
BU233962	2.626067046	1.39290375
CN229585	2.619008774	1.389020893
CR391180	2.618909458	1.388966183
BM488196.1	2.617439571	1.38815623
TC223354	2.617203595	1.388026158
CR523336	2.617100292	1.387969212
CR352845	2.616414717	1.387591235
CR391033	2.616136931	1.387438055
CO757296	2.610985327	1.38459435
CR391153	2.608727432	1.383346215
BX930271	2.608268028	1.38309213
TC207177	2.607607941	1.382726974
AJ443771	2.605599021	1.381615083
TC201014	2.603081016	1.380220214
CO773597.1	2.599462058	1.378213098
BU229963	2.598013304	1.377408819

NTS	2.594407497	1.375405098
BU480632	2.593534914	1.374919792
CR354310	2.592952899	1.374596
CR353362	2.575631307	1.364926091
AJ450643.1	2.574496559	1.364290342
BU463302	2.570010325	1.361774156
RCJMB04_3c14	2.569308603	1.361380185
BX933975	2.566191725	1.359628961
AJ719902	2.564053339	1.358426274
CR523720	2.560455838	1.356400676
CR389220	2.549890057	1.350435044
BX935537	2.548466665	1.349629483
CR353481	2.548277397	1.349522333
BU246292	2.540423383	1.345068954
CR523258	2.537320048	1.343305507
CR386009	2.53701536	1.343132254
CR390588	2.536569721	1.342878815
CR388507	2.53418774	1.341523407
AJ442718	2.522393113	1.334793136
CR353306	2.517945265	1.332246922
U43396	2.516396986	1.331359539
BX931842	2.515718553	1.330970529
TC202313	2.513030185	1.329428
CR382437	2.506040029	1.325409459
BU220021.1	2.50384976	1.324147998
CR407288	2.502640828	1.323451254
BX934900	2.501378293	1.322723258
TC211945	2.493982473	1.318451327
BX933660	2.492493539	1.317589765
BU390708.1	2.488769686	1.315432728
LOC421487	2.48525165	1.313391943
CO421911	2.483830445	1.312566693
TC225175	2.483315488	1.312267558
BU409199	2.483236411	1.312221617
CR391057	2.482094319	1.311557939
CR385326	2.47140136	1.305329326
BU349333	2.4707628	1.304956515
CK612856	2.470164125	1.304606902
SLC24A2	2.468273221	1.3035021
BU451798	2.467266344	1.302913464

CR388753	2.466042445	1.302197631
AF053401	2.465826192	1.302071113
CO768248	2.4648266	1.301486157
TC186436	2.463188157	1.300526836
TC195838	2.458353532	1.297692402
BX935610	2.456532185	1.296623141
CR390520	2.454625037	1.295502658
CR388514	2.451305251	1.293550148
DR430202	2.450417999	1.293027869
TC227258	2.450375863	1.293003061
BI394628	2.447039687	1.2910375
TC207806	2.4468122	1.290903374
BU287063	2.438528358	1.28601075
BX934417	2.438247085	1.285844332
BU351409	2.435892497	1.284450464
TC202650	2.434975489	1.28390725
RCJMB04_8f10	2.434324643	1.283521579
AJ719724	2.426898614	1.27911384
LOC420928	2.424853064	1.277897328
CF250694	2.421832979	1.276099374
CR391664	2.41992119	1.274960064
BX931927	2.4182789	1.27398064
CR524053	2.416348427	1.2728285
BU445218	2.412953871	1.270800335
CR385522	2.409612782	1.268801328
CR385971	2.409290254	1.26860821
CR406889	2.398614143	1.262201095
BU421292	2.398430556	1.262090668
BU364681	2.396891036	1.261164325
BU306495	2.396831219	1.261128321
LOC420153	2.395483168	1.260316676
BX934875.2	2.394615259	1.259793878
CR388873	2.390613269	1.257380763
CR352665	2.390143895	1.257097476
RCJMB04_1a11	2.389764555	1.256868488
AJ719928	2.38924715	1.256556098
TC198418	2.382824701	1.25267282
TC223799	2.382794228	1.25265437
AB167977	2.381002823	1.251569331

BU218129	2.376678245	1.248946604
TC202228	2.375582088	1.24828106
BX271667	2.371256908	1.245651976
TC225586	2.370657075	1.245286986
LOC418249	2.370573892	1.245236364
CR353316	2.368553507	1.244006264
BU385348	2.368288766	1.243845
BU394314	2.367837524	1.243570089
AF043432	2.366466713	1.242734629
CO763867	2.36377518	1.241092826
TC204650	2.361374725	1.239627
CR352419	2.360461578	1.239069
TC226608	2.357445412	1.237224365
BU339047	2.356338547	1.236546834
CR407152	2.351836114	1.233787531
BX934934	2.350016195	1.232670699
ENSGALT00000002107.2	2.349327325	1.232247734
TC215664	2.347966757	1.231411983
BU336892	2.347668599	1.23122877
CV861257	2.344283916	1.229147305
TC197611	2.343438856	1.228627153
CR352928	2.338415607	1.225531363
CR406110	2.335072	1.223467035
BX933451	2.330159967	1.220429
AJ719379	2.329935501	1.220290018
AF427472	2.329828886	1.220224
BU304245	2.327983713	1.219080965
RCJMB04_2h7	2.325390838	1.217473216
AM064873.1	2.324080387	1.216659971
CR387667	2.323867908	1.216528066
TC201574	2.320548632	1.214465933
BU376006	2.320203935	1.214251617
TC223400	2.31978527	1.213991269
TC190285	2.318423183	1.213143927
BU373407	2.31642885	1.21190237
TC215057	2.31431782	1.210587
BX932650	2.311961205	1.20911719
ABCB7	2.31165576	1.208926575
BU243264	2.310920565	1.20846767
BU276481	2.310303727	1.20808253

BM427473	2.309844105	1.207795485
TC227297	2.309722451	1.2077195
BX930770	2.309651434	1.207675141
AF167296	2.305672701	1.205187731
CF257992	2.304499716	1.20445359
BU313448	2.304228736	1.204283937
CN223874	2.301227772	1.202403787
CD764914	2.299976743	1.201619273
TC186047	2.299550074	1.201351614
CV861612	2.297758593	1.200227233
BU302385	2.29537537	1.198730101
TC207230	2.294762683	1.198344963
BU228537	2.286406441	1.193081886
NFASC	2.282965548	1.190909088
CR353086	2.282075232	1.190346353
CO761770	2.279936273	1.1889935
AJ251273	2.277448193	1.187418236
CR391170	2.276964816	1.187111999
CR390117	2.2769273	1.187088228
BU105518	2.273675901	1.185026622
BU351210	2.273232081	1.184744981
AJ720009	2.271407492	1.18358655
TC211110	2.271157424	1.183427709
CR385255	2.270157615	1.182792466
BX935306	2.26710276	1.180849785
BU469217	2.266434236	1.1804243
COLEC12	2.264931979	1.179467724
TC205803	2.264009102	1.178879758
BX934087	2.260756068	1.176805336
BX931113	2.2589205	1.175633498
AJ720303	2.25571394	1.173584123
BU113669	2.254185935	1.17260652
AM071896.1	2.251980553	1.171194369
CR353337	2.248792075	1.169150275
BU452676	2.247760544	1.168488352
CR391587	2.247068263	1.168043952
TC186192	2.245908265	1.167299001
TC219879	2.245704777	1.167168282
AJ719634	2.240905794	1.164082
BX930128	2.237551459	1.161920862
BX935178	2.236256676	1.161085789

SLC7A6OS	2.234319616	1.159835576
CR353323	2.233318747	1.159189172
BU488540	2.233151262	1.159080975
CR388523	2.231775253	1.15819175
TC226515	2.230520326	1.157380295
AF373778	2.227670309	1.155535732
RAMP3	2.2275954	1.155487218
CR387310	2.225985729	1.154444344
CR385236	2.221931361	1.15181425
BU459894	2.220322127	1.150769
D43697	2.219307613	1.15010965
BU426292	2.215304575	1.147505064
AB083370	2.214787485	1.147168275
BU264054	2.214526525	1.146998278
CR385700	2.213176868	1.14611875
TC208890	2.206816317	1.141966553
BU127719.1	2.206630056	1.141844781
AF380350	2.206217399	1.14157496
BX931735	2.205944933	1.141396777
TC223239	2.205071425	1.140825387
BU382793	2.20477984	1.140634602
CR352693	2.204704802	1.1405855
SDSL	2.203842789	1.140021313
CR385693	2.20224001	1.138971709
CR386489	2.201904112	1.138751644
WBP2	2.201354353	1.138391395
L21719	2.199718475	1.137318896
TC188871	2.198700018	1.136650782
BU472358	2.196834623	1.135426268
BU313215	2.192539441	1.132602795
TC206052	2.190427554	1.1312125
BU303572	2.18853187	1.129963393
BU452988.1	2.18668199	1.128743424
BU230639	2.185103902	1.127701882
TC227161	2.182165255	1.125760361
CNTN4	2.179137031	1.123756921
BX933950	2.178552223	1.123369697
TC207996	2.174885575	1.1209395
TC218946	2.170355787	1.117931563
U77642	2.169208354	1.117168631
BU202212	2.163006411	1.113037941

AJ720962	2.161678933	1.11215226
BX950556	2.161644348	1.112129178
AJ851403	2.160673255	1.111480919
CV862149	2.160514449	1.11137488
CR406082	2.160376437	1.111282718
AB124569	2.157551875	1.109395247
CR385561	2.157289217	1.109219604
TC206065	2.156865553	1.10893625
BU302284	2.156346621	1.108589102
TC200776	2.154981901	1.107675753
BU483614	2.152901944	1.106282612
BX936016	2.151556067	1.105380436
BU294348	2.14936724	1.103912002
BU143619	2.149259147	1.103839446
AJ719806	2.149212852	1.10380837
CR385282	2.147382036	1.102578881
CB016517	2.147230263	1.10247691
XM_416059	2.145808988	1.101521658
AJ719455	2.141807716	1.098828966
BX935712	2.139724154	1.097424821
CR523686	2.13792788	1.096213187
LOC420227	2.136803499	1.095454243
INPP5F	2.134555437	1.093935632
CR386456	2.134031109	1.093581207
CR385592	2.13399363	1.093555869
BU475165	2.132718127	1.092693303
INHBA	2.127992026	1.089492745
M34096	2.126120836	1.088223593
BU396717	2.125780579	1.087992691
BU243657	2.123150661	1.08620675
BM426715	2.123002105	1.086105802
CR353996	2.121872626	1.085338055
CO503248.1	2.121400621	1.085017096
AJ719865	2.119699482	1.083859743
TC207503	2.117102536	1.082091144
CD216048	2.116809365	1.081891349
AJ720485	2.115449726	1.0809644
BU314764	2.114602692	1.080386624
BU424877	2.112844357	1.079186495
BX261488	2.108503413	1.076219357
TC202179	2.107420802	1.075478415

BU203200	2.106816092	1.075064384
CR386371.1	2.105021996	1.073835309
TC190860	2.104737227	1.073640126
BM491348	2.104648967	1.073579627
BX935190	2.103556848	1.072830807
PHLDA2	2.103452445	1.072759202
CR386490	2.101969142	1.07174149
AJ720657	2.099477124	1.070030069
BU461716	2.098120099	1.069097262
BX934074	2.097716233	1.068819532
BX929722	2.097676987	1.06879254
BX934261	2.096551337	1.068018157
AJ851592	2.096211028	1.067783962
CR389846	2.096139197	1.067734524
BX930076	2.090593491	1.063912562
TC207124	2.089401803	1.063089957
AB031025	2.083511046	1.059016749
TC211068	2.080224342	1.056739124
TC212635	2.077192245	1.054634745
TC221654	2.076547434	1.054186827
BX950834	2.076215579	1.05395625
CR353053	2.076076247	1.05385943
AF406812	2.075603288	1.053530726
RGS3	2.075213707	1.053259914
CR523071	2.074991298	1.053105286
BU138627	2.074841719	1.053001284
BX950629	2.074838607	1.05299912
CR523627	2.074450187	1.052729015
AJ719980	2.0730215	1.051735079
CR387164	2.071479504	1.050661546
CR354113	2.068996403	1.048931137
BU338225	2.067171421	1.04765803
X89248	2.064929253	1.046092354
BU291774	2.064410597	1.045729942
LOC416073	2.064307727	1.04565805
TC204154	2.060235771	1.042809447
CR406678	2.06014876	1.042748516
CR353846	2.059602134	1.04236567
RCJMB04_14i19	2.058986206	1.041934165
BX279056	2.057921786	1.041188152

GAL12	2.056814785	1.040411886
CD737580	2.056746525	1.040364006
CR406369	2.054972368	1.039118995
BU436163	2.053892797	1.038360882
CR385846	2.053132903	1.037827018
CO767385	2.05215972	1.037143021
AM065594.1	2.051927978	1.036980094
BU237399	2.051909417	1.036967043
L39766	2.05102017	1.036341679
BU415681	2.049441415	1.03523075
MOSPD2	2.047256403	1.0336918
CR389396	2.046835946	1.033395475
BU218523	2.046283015	1.033005694
CR391210	2.046111241	1.032884582
CR390913	2.045562687	1.03249775
BU217692	2.044463089	1.031722017
BX933308	2.044377355	1.031661516
BU489399	2.043797668	1.03125238
CN606337	2.043673103	1.031164448
BU460377	2.043209304	1.030837
CR406530	2.040440653	1.02888075
BU473486	2.040206845	1.028715426
BU225152	2.039825753	1.028445919
AJ720557	2.039466031	1.028191478
TC227370	2.03859361	1.027574205
BX933889	2.038469869	1.027486632
AY237249	2.038337452	1.027392913
TC188062	2.037589908	1.026863719
BU437475	2.036985707	1.026435857
TC193829	2.035828043	1.025615709
LOC416518	2.035401196	1.025313191
BU451593	2.035347114	1.025274857
BU241522	2.03311207	1.023689742
BU235277	2.032487111	1.023246204
BX950669	2.031934357	1.022853795
CR354117	2.030902886	1.022121254
BX931425	2.029452991	1.021090923
DN854402	2.02932861	1.0210025
AB002585	2.028124958	1.020146544
CR385263	2.027743045	1.019874846
BU448010	2.025956616	1.018603281

BU229315	2.025764131	1.018466205
TC227372	2.025262624	1.018109
MSTO1	2.022465711	1.016115243
CR390946	2.022457309	1.01610925
U02881	2.022105543	1.0158583
BU302509	2.021776217	1.015623319
CR353322	2.020618764	1.01479715
BX935071	2.018893202	1.013564595
AF216970	2.018103228	1.012999972
CHIC2	2.016719848	1.012010686
BU200000	2.016667168	1.011973
M32293	2.015966904	1.011471955
BU271833	2.015607878	1.011215
CR387538	2.015452072	1.011103476
CR353866	2.014287751	1.010269794
BU309556.1	2.013214396	1.009500819
CR385759	2.012037853	1.008657447
CD729265	2.011790652	1.008480185
CR390363	2.011536669	1.008298037
SLC30A6	2.009954469	1.00716282
AJ720887	2.009792978	1.007046902
BG712426	2.009256077	1.006661445
BX932772	2.008487015	1.006109134
TC223160	2.007854879	1.005655
CR385092	2.00487275	1.003510671
BX933579	2.004347719	1.003132812
AJ720866	2.004065825	1.002929896
CR389713	2.003817167	1.00275088
BU324362.1	2.003686046	1.002656473
BU447021	2.003504461	1.002525722
BX936153	2.003096728	1.002232089
CR386164	2.002180591	1.001572107
TC189209	2.001613951	1.00116375
TC216140	2.001129212	1.000814324
BX930456	0.499950894	-1.000141697
LOC423166	0.499872479	-1.000367996
TRIM55	0.499723953	-1.000796724
CO759418	0.499330026	-1.001934433
LOC418733	0.499268582	-1.00211197
CR385268	0.499126894	-1.002521453
TC188049	0.498616808	-1.003996579

CR523476	0.498481074	-1.004389364
CR386414	0.49821257	-1.005166672
TC211590	0.498063899	-1.00559725
PMP2	0.497636507	-1.006835768
CN223346	0.497583339	-1.006989915
AJ720320	0.497230769	-1.008012521
BU485854	0.497170847	-1.008186394
RCJMB04_10e24	0.496977959	-1.008746226
AF253514	0.496877351	-1.009038314
BM426076	0.496712956	-1.009515716
CO766327	0.496682638	-1.009603777
TC203029	0.49648416	-1.010180405
TC216735	0.495976682	-1.0116558
BU216764	0.495766944	-1.012266015
BU130295	0.495711036	-1.012428716
BU433061	0.495621778	-1.012688514
BU339908	0.495556777	-1.012877736
TC198219	0.49544444	-1.013204814
TC219154	0.495434902	-1.01323259
BU320564	0.49518351	-1.013964821
TC197808	0.494877598	-1.01485636
BX934029	0.494855612	-1.014920455
CR733305	0.494841962	-1.01496025
LOC424657	0.494694666	-1.01538975
AJ719471	0.494651436	-1.01551583
AJ449433	0.49456577	-1.015765703
CR406729	0.49453489	-1.015855787
CR389443	0.494484858	-1.01600175
BU139451	0.494466617	-1.016054973
RCP9	0.494452266	-1.016096844
RCJMB04_1a13	0.4942265	-1.016755725
BU303195.1	0.494055845	-1.017253971
CR407525	0.493723105	-1.018225934
BU224391	0.493401065	-1.019167266
BU224177	0.493196124	-1.019766632
AJ720161	0.493125923	-1.019972
CO764184	0.492768968	-1.02101669
CR389944	0.492578758	-1.021573682
CR353200	0.492529486	-1.021718

CR406615	0.492114012	-1.0229355
CO764197	0.491819645	-1.023798732
CO767654	0.491519444	-1.024679606
AJ719947	0.491194656	-1.025633229
TC202170	0.490918002	-1.026446023
ENSGALT00000033345.1	0.490614274	-1.027338886
BU277485	0.490488122	-1.027709897
CR352842	0.490454763	-1.027808019
BU276382	0.489664996	-1.030133027
TC216367	0.489623879	-1.030254174
TC223562	0.489586284	-1.030364953
CB017570	0.489522404	-1.030553205
BM491412	0.489490432	-1.030647434
U20216	0.489432338	-1.030818669
BX930260	0.489137393	-1.031688335
BU423658	0.48910399	-1.031786861
CR387354	0.488867537	-1.032484489
BU274618	0.488812927	-1.032645655
BU465509	0.488766717	-1.032782048
CR386136	0.488743537	-1.032850469
FBLN5	0.488721429	-1.03291573
AF294794	0.488649996	-1.033126616
BU389691	0.488398592	-1.033869055
CR390264	0.488363961	-1.033971354
PPP3CA	0.488207377	-1.034434
CR385188	0.487544788	-1.036393337
TC191003	0.486894917	-1.038317654
SPG7	0.486794134	-1.03861631
BX931232	0.486655423	-1.039027463
BU374510	0.486472325	-1.03957036
BU466859	0.486255776	-1.040212707
CR387786	0.486253942	-1.040218148
AJ720696	0.48623803	-1.040265359
RWDD3	0.486206572	-1.0403587
TC192752	0.486147517	-1.040533942
AF222990	0.485478564	-1.0425205
TC196276	0.485350954	-1.042899766
CR523254	0.485244857	-1.043215173
CN209883	0.48515973	-1.043468289
CR523505	0.48496961	-1.04403375

CR386560	0.484860141	-1.044359437
TC223779	0.484856166	-1.044371263
CR523173	0.484409853	-1.045699884
TC191488	0.484236251	-1.046217008
CR406890	0.484081655	-1.046677674
CO767666	0.48393142	-1.047125483
CR386776	0.483907052	-1.047198132
TC194483	0.483779629	-1.047578073
TC186423	0.48328315	-1.049059401
CR354244	0.483139465	-1.049488393
TC195213	0.483060064	-1.049725509
CD763590	0.482999471	-1.049906485
AF245511	0.482876614	-1.0502735
AF208401	0.482511058	-1.051366089
AJ393261	0.482422535	-1.051630794
BX935571	0.482056541	-1.052725722
CR386271	0.481966985	-1.052993769
TC214893	0.481952341	-1.053037605
TC224586	0.481939858	-1.053074973
TC218211	0.481932154	-1.053098035
BQ038076	0.481930072	-1.053104269
THBS2	0.481884316	-1.05324125
BX936212	0.481795307	-1.053507755
TC226456	0.481780111	-1.053553258
CR406894	0.481695706	-1.053806033
CR406764	0.481505627	-1.054375437
BX931494	0.481466995	-1.054491192
BX262827	0.481411911	-1.054656257
BX933970	0.48110668	-1.055571265
CR389388	0.480912097	-1.056154879
M24282	0.480862058	-1.056305
BU277690	0.480658909	-1.05691462
BU371222	0.480646722	-1.056951199
CR354291	0.480526838	-1.057311085
BX935638	0.480516073	-1.057343405
S78477	0.480154433	-1.058429596
BX931854	0.479958609	-1.059018099
CR353382	0.479641786	-1.059970745
TC198910	0.479602356	-1.06008935
BU332793	0.479462703	-1.0605095
TC218232	0.47920597	-1.061282213

AF465268	0.479170536	-1.061388895
BU358463	0.47910337	-1.061591132
CR385655	0.47900208	-1.061896174
BU380097.1	0.478989547	-1.061933922
BX950717	0.478671152	-1.062893232
TLR7	0.478492872	-1.063430663
CR352822	0.478389125	-1.0637435
CR386540	0.478150166	-1.064464319
AF144636	0.478006953	-1.064896493
PPP1R3B	0.477967942	-1.065014237
BU217611	0.477516859	-1.066376426
BX261988	0.477359755	-1.066851152
CV860424	0.477332343	-1.066934
TC215291	0.477320256	-1.066970535
AJ398827.1	0.477129858	-1.067546123
CR386575	0.477025025	-1.067863143
AJ720414	0.476778547	-1.068608773
CO507473	0.476766202	-1.068646129
CR385139	0.476487748	-1.069488978
BU389236	0.476263381	-1.070168469
CR353726	0.476242285	-1.070232374
TC224545	0.476080736	-1.070721842
BU114438	0.475687215	-1.071914845
AJ720793	0.475675541	-1.07195025
TC225266	0.475604324	-1.072166265
CR407028	0.475585684	-1.072222807
CO765459	0.475307341	-1.07306741
TC190734	0.475207626	-1.073370106
AJ721051	0.475063089	-1.073808978
CD735765	0.474928929	-1.074216459
AY388624	0.474806223	-1.07458925
BU245711	0.474569384	-1.075309063
TC225333	0.474347712	-1.075983107
EXOC4	0.474293952	-1.076146622
CR524391	0.47422556	-1.07635467
SH3PXD2A	0.473983258	-1.077091993
TC188045	0.473730925	-1.077860242
BX935878	0.473632505	-1.07816
BU286076	0.473509896	-1.078533519
TC195585	0.473059087	-1.079907703
TC207984	0.472884169	-1.080441251

BU360659	0.472846499	-1.080556179
BU352846	0.472606013	-1.081290109
EGFL7	0.472492119	-1.081637828
BU213138	0.47246381	-1.081724271
CR524449	0.472379024	-1.08198319
CR385866	0.472300281	-1.082223702
TC194664	0.472284161	-1.082272941
TC207011	0.471937819	-1.083331309
BU465980.1	0.47188668	-1.083487645
AJ295030	0.471756605	-1.083885379
RCJMB04_23o9	0.4712301	-1.0854964
BU375946	0.470117894	-1.088905501
HAS2	0.470051329	-1.08910979
CD760772	0.469381128	-1.091168258
CR387763	0.469277355	-1.09148725
TC226969	0.4691565	-1.091858842
BU241454	0.468855593	-1.092784453
BU215169	0.46885097	-1.092798678
L02270	0.468704446	-1.093249616
CN226123	0.468692057	-1.09328775
BU402278	0.4681722	-1.094888825
BU202455	0.468170114	-1.094895254
AJ721104	0.468129229	-1.095021248
BU477136	0.468107124	-1.095089375
BX932538	0.467816234	-1.095986168
TC223106	0.467735321	-1.096235716
AJ719353	0.467525891	-1.096881834
BU326392	0.467345773	-1.097437751
CR407154	0.467261984	-1.097696428
BX931872	0.466959053	-1.098632047
BX930762	0.466937948	-1.098697253
AY714074	0.46683013	-1.099030418
TC205527	0.466350442	-1.100513611
AJ720481	0.466224149	-1.100904361
CO772107.1	0.465957549	-1.101729572
BU248677	0.465701628	-1.102522171
CN209965	0.465321757	-1.103699449
AF224314	0.464975299	-1.104774019
TC195503	0.464867557	-1.10510835
CR388895	0.464729116	-1.105538061

BU291832	0.464333879	-1.106765547
BU402592	0.463744081	-1.108599226
BX950818	0.463184524	-1.110341044
L08165	0.462828947	-1.111448996
BU409410	0.462816821	-1.111486794
BU476318	0.462361868	-1.112905675
TC202520	0.462325187	-1.113020133
CR352447	0.462240122	-1.113285606
BU121409	0.462040268	-1.113909503
U85022	0.461885248	-1.114393626
TC194300	0.461738208	-1.114852976
CR352929	0.461713548	-1.114930029
CD732070	0.461651408	-1.115124209
CR352817	0.461421718	-1.115842184
TC227524	0.461290616	-1.11625215
BX270333.4	0.461254631	-1.116364699
CR405764	0.461208328	-1.11650953
BX930043	0.461030689	-1.117065306
DN930251	0.460445587	-1.118897419
BU255508	0.460238854	-1.119545311
CR354098	0.46000518	-1.120277988
TC207774	0.459833189	-1.120817498
CR385921	0.459750962	-1.1210755
TC193311	0.459560435	-1.121673497
CR352672	0.459281841	-1.122548353
ATG10	0.459211243	-1.12277013
TC211881	0.459160396	-1.122929885
CR390395	0.459085567	-1.123165019
LSAMP	0.45887878	-1.123815
AJ719698	0.458871072	-1.123839236
LOC429138	0.458715368	-1.124328855
SEMA3A	0.458639469	-1.124567581
TC209573	0.45858096	-1.124751638
CN232164	0.458365561	-1.125429442
M10013	0.45835061	-1.1254765
CF250524	0.458350452	-1.125477
CR353214	0.456903289	-1.130039268
BM426696	0.456869867	-1.130144804
CR406196	0.456626311	-1.130914106
M35043	0.456587713	-1.131036058
BQ037430	0.456513763	-1.131269738

TC211965	0.455954215	-1.133039133
BX930280	0.455819769	-1.133464598
BX276973	0.455655014	-1.133986151
CR391007	0.455631592	-1.134060314
TC217965	0.455145301	-1.135600907
BU247182	0.455033713	-1.135954657
BX932547	0.455008782	-1.136033703
TC206703	0.454809873	-1.136664523
AJ719708	0.454493204	-1.137669371
CO756815	0.454492306	-1.137672223
U27708	0.454477298	-1.137719863
CR522935	0.454356411	-1.138103658
DR426369.1	0.454151505	-1.138754435
AJ720558	0.454130999	-1.138819576
PDE5A	0.454122878	-1.138845374
CR405854	0.453522417	-1.140754233
SPON1	0.453414728	-1.141096842
BU462227	0.453402644	-1.141135291
CR386300	0.453275144	-1.141541045
CR732866	0.45301101	-1.14238198
AJ720736	0.452908517	-1.142708427
TC222249	0.452525539	-1.143928879
AJ720968	0.45248723	-1.144051019
TC224301	0.452437073	-1.144210944
BX272499	0.452210688	-1.144933003
TC213432	0.452209052	-1.144938224
CR388715	0.452194529	-1.144984558
TC224021	0.45215666	-1.145105382
CR387021	0.452142187	-1.14515156
TC192570	0.452066683	-1.1453925
BU471460	0.451618009	-1.146825078
ENSGALT00000002596.2	0.451603631	-1.146871009
AB032197	0.450972776	-1.14888775
CR390251	0.450262661	-1.151161251
CR386078	0.450075494	-1.151761082
PPTX	0.450074963	-1.151762781
CN227059	0.449516136	-1.153555189
BX931558	0.449408125	-1.153901889
BU133261	0.449277191	-1.154322275
EBF	0.449205788	-1.154551579

BX935155	0.449133674	-1.154783202
TC187902	0.448870926	-1.15562744
CR407258	0.44861795	-1.15644075
ENSGALT00000012833.1	0.448606895	-1.156476299
TC187862	0.448098548	-1.158112044
LOC419762	0.448047739	-1.158275635
TC203923	0.447242773	-1.160869925
CR391519	0.447034163	-1.161543007
AJ720816	0.44671817	-1.162563158
BU462673	0.44666663	-1.162729619
CR353850	0.446561315	-1.163069817
BU310709	0.446319297	-1.16385191
CR389849	0.44629451	-1.163932034
M29076	0.446116148	-1.164508724
BX929995	0.446105657	-1.164542651
M22154	0.445860269	-1.16533645
GLUL	0.445817715	-1.16547415
CR391405	0.445433019	-1.166719588
CN234101	0.445264893	-1.167264229
AJ721108	0.44517077	-1.167569228
BU465112	0.444541091	-1.169611316
RCJMB04_9m19	0.444433509	-1.1699605
BX950700	0.443765371	-1.172131003
TC195237	0.443447522	-1.173164709
TC205763	0.44312092	-1.174227657
BU356045	0.442762331	-1.175395609
PITX1	0.442662346	-1.175721434
BU117677	0.442444023	-1.176433153
BX933868	0.442428245	-1.176484603
BX935026	0.442374194	-1.176660867
TC206002	0.442196199	-1.177241472
BU292735	0.441677133	-1.178935955
BU465190	0.441649278	-1.179026942
BU303861	0.44163318	-1.179079527
BX932207	0.441082801	-1.180878588
AJ131110	0.440964124	-1.18126681
BU368502	0.440817795	-1.18174563
CN210453	0.440677149	-1.182206007
CR407225	0.440464304	-1.182902988

TC202136	0.440442037	-1.182975925
BU278422	0.440152994	-1.183923013
CV858675	0.439992008	-1.184450777
BX950646	0.439928516	-1.184658974
BU206650	0.439798568	-1.185085189
BX279178	0.439776846	-1.185156445
BU455881	0.439057596	-1.187517888
LOC421052	0.438868097	-1.188140696
CN226691	0.438847721	-1.188207681
CR391618	0.438761028	-1.188492706
BU213515	0.438660123	-1.188824532
CV892777	0.438548109	-1.189192979
BU381248	0.438279689	-1.190076271
CR338949	0.438069641	-1.190767859
CR523818	0.437935847	-1.19120855
GHR	0.437597664	-1.192323058
BU119118	0.43753688	-1.192523467
CN210324	0.437525436	-1.192561202
CV860562	0.437048955	-1.194133206
PSPH	0.436922089	-1.194552052
BX950437	0.436921912	-1.194552635
CR352567	0.436907019	-1.194601811
CR388846	0.436843829	-1.194810486
CR406008	0.436730441	-1.195185
BU411355	0.436714371	-1.19523809
M94894	0.436699616	-1.195286831
CO766464	0.436691753	-1.195312808
CR523467	0.436672885	-1.195375146
BU350598	0.436443611	-1.196132825
CN384348	0.436404659	-1.196261592
BX950638	0.435801013	-1.198258546
BX950292	0.435454164	-1.199407228
TC207295	0.435070903	-1.200677562
WNT2B	0.434983854	-1.200966244
CR386281	0.434903435	-1.20123299
TEAD1	0.434894793	-1.201261658
AF116856	0.434791079	-1.201605755
TC193763	0.434742805	-1.201765942
BU396107	0.434663646	-1.202028656
CR389693	0.434662469	-1.202032563
MYH6	0.434653977	-1.20206075

BU135931	0.434508643	-1.202543222
XM_420245	0.434446234	-1.202750452
AF472618	0.434263393	-1.20335775
CO761287	0.434213692	-1.203522875
BU315749	0.433893934	-1.204585678
CO764320	0.433530058	-1.205796071
BU390536	0.433453609	-1.2060505
TC226848	0.433286627	-1.206606386
TC195680	0.432885924	-1.207941207
CR406658	0.4328085	-1.208199261
TC223199	0.432789991	-1.208260961
BU343308	0.43263519	-1.208777079
CR523186	0.432592258	-1.20892025
BX934389	0.432501469	-1.209223062
BU333083	0.432482863	-1.209285126
BU404514	0.432227494	-1.21013725
ENSGALT00000009597.2	0.432067552	-1.210671206
CR406675	0.431909354	-1.211199534
ENSGALT00000008514.1	0.431249778	-1.21340438
CR353414	0.430803847	-1.214896961
BU202234.1	0.430538557	-1.215785649
CR387585	0.430511235	-1.215877207
CR524190	0.430503053	-1.215904627
CR352647	0.43036719	-1.216359999
BU253122	0.430224787	-1.216837448
BU326858	0.430115613	-1.217203594
CR390540	0.429856247	-1.218073822
MEIS2	0.429827207	-1.21817129
CR353501	0.429682165	-1.218658198
TC207844	0.429483095	-1.21932675
BX273199	0.429009032	-1.220920075
AJ720202	0.428983523	-1.22100586
AL588023	0.428948759	-1.221122776
TC199317	0.428702523	-1.221951187
TC216756	0.428649843	-1.222128479
BX934090	0.42852417	-1.222551516
AJ720654	0.428385558	-1.22301825
AB161354	0.428092156	-1.224006692
PPP1R1C	0.428029228	-1.224218782

BU387850	0.427933916	-1.22454007
FAM20C	0.427853417	-1.224811483
CAB39L	0.42780164	-1.22498608
BU469992	0.427719432	-1.225263341
BX930231	0.427381517	-1.226403579
BX933588	0.427242997	-1.22687125
BX936064	0.427238916	-1.22688503
M19496	0.427215667	-1.226963541
BU478480	0.427208578	-1.22698748
BU389217	0.426987478	-1.227734334
TC208825	0.426961208	-1.227823098
CR524111	0.426710055	-1.22867199
CR523090	0.426588688	-1.229082385
TC202085	0.426457446	-1.229526306
AJ719629	0.426294059	-1.230079144
CR406076.1	0.426293793	-1.230080047
BX271748	0.425995555	-1.231089719
BU432366.1	0.425954979	-1.23122714
AB108842	0.425908031	-1.231386161
MAP1A	0.425762841	-1.231878052
WT1	0.425467481	-1.232879226
BI066544	0.42518916	-1.233823278
CR391555	0.424590567	-1.235855775
BU232348	0.42429222	-1.23686987
BX933437	0.424270242	-1.236944604
TC195342	0.424200517	-1.237181717
BU269342	0.42401767	-1.237803709
TC224567	0.423923391	-1.23812452
CV856945	0.423538404	-1.239435304
AF062392	0.423430107	-1.239804244
CR389732	0.42334695	-1.240087601
TC224375	0.422898337	-1.241617208
BU441119	0.42287634	-1.241692253
CR524390	0.42285782	-1.241755436
BI391130	0.4228053	-1.241934633
TC218624	0.422757592	-1.242097431
REEP1	0.422515365	-1.24292429
BX935863	0.422409165	-1.243286956
AF050131	0.422038912	-1.244552072
CN226760	0.422002606	-1.244676186
DN928754.1	0.421787148	-1.245412957

BX261224.4	0.421519356	-1.246329215
TC213571	0.421314799	-1.247029502
BU405197	0.42111165	-1.247725306
BU390859	0.421094219	-1.247785025
TC186950	0.420884224	-1.24850466
TC199227	0.420785744	-1.248842267
BX929970	0.420417987	-1.250103701
BU106671	0.420361648	-1.250297045
BU447247	0.420310383	-1.250473
CR390097	0.420195647	-1.250866878
TC200815	0.420042671	-1.2513922
CF250950	0.4198851	-1.2519335
CR386224	0.419369975	-1.25370452
TC203619	0.41936061	-1.253736737
TC222677	0.419284789	-1.253997601
TC220592	0.419226516	-1.254198126
CR385281	0.419218956	-1.254224141
BU391632	0.418790556	-1.255699187
CR352491	0.418610578	-1.256319326
MAPKAPK5	0.418569284	-1.25646165
TC225942	0.418361133	-1.257179268
RAPSN	0.418308834	-1.257359631
BU142949	0.41824425	-1.257582387
BU241997	0.417864147	-1.258894113
BU441936	0.417813603	-1.259068632
TC226615	0.417616913	-1.259747954
FGF13	0.417330384	-1.260738134
PHACTR1	0.417241311	-1.261046089
BU465302	0.417165704	-1.261307539
BX277859	0.416866415	-1.262342949
SERPINF1	0.416851263	-1.262395389
CR354195	0.416832733	-1.262459522
TC205246	0.416826228	-1.262482037
J03737	0.416698694	-1.262923517
LOC415782	0.416643153	-1.263115822
CR386730	0.416510104	-1.263576601
CR385239	0.41649296	-1.263635985
TBC1D14	0.416477511	-1.2636895
BU449952	0.416394226	-1.263978031
MYOT	0.416314549	-1.264254119
CR382435	0.416158934	-1.264793487

AJ719347	0.416003755	-1.265331545
CD764090	0.415912066	-1.265649555
BU393144	0.415856761	-1.265841407
CR524171	0.415461026	-1.267214949
CR352683	0.415309439	-1.267741434
CR389027	0.415298487	-1.267779479
BU377412	0.415165095	-1.268242942
TC214956	0.414965994	-1.268934982
CF250409	0.414320925	-1.271179409
AJ720846	0.414243346	-1.27144957
TC224014	0.414234587	-1.271480078
AL584098	0.41422657	-1.271508
TC197013	0.41416764	-1.271713259
BU350117	0.414042343	-1.272149779
PTPRG	0.413553807	-1.273853046
BX931252	0.413548659	-1.273871003
BU471733	0.413485624	-1.274090925
AJ719733	0.412750526	-1.27665804
BU409113	0.412016641	-1.279225486
CR385766.1	0.412001241	-1.279279412
CF253380	0.411842877	-1.279834059
AJ719608	0.411115374	-1.282384771
AF285876	0.410982949	-1.282849556
CR387154	0.410963999	-1.282916079
BX263106	0.410875132	-1.28322808
TC202710	0.410774145	-1.283582716
TC226293	0.410561377	-1.284330181
AJ851551	0.410454895	-1.284704402
CO769894	0.410440188	-1.284756098
BU474543	0.410220928	-1.285527
BU479930	0.410005857	-1.286283574
BX950647	0.409828286	-1.286908535
BU280833	0.40946293	-1.28819525
ST6GALNAC1	0.409249804	-1.28894637
CR523032	0.409088421	-1.28951539
CR524147	0.409068105	-1.289587042
CR390166	0.408991928	-1.289855724
PRRX1	0.408971913	-1.289926328
BU405757	0.408717431	-1.290824322
BX936224	0.408572589	-1.291335678
RCJMB04_12i1	0.408200779	-1.292649161

3		
CR387712	0.407841589	-1.293919194
TC217725	0.407684603	-1.294474622
CV861566	0.407029022	-1.296796429
BX260306	0.406965529	-1.297021494
BU110178	0.406960076	-1.297040825
TC216688	0.406900361	-1.297252534
DT659901	0.406853907	-1.29741725
U91630	0.406454326	-1.29883485
CR391266	0.406351005	-1.299201632
AJ719931	0.406200717	-1.29973531
CR523439	0.406134745	-1.29996964
DN854854.1	0.406125879	-1.300001133
BQ038996.2	0.406018691	-1.300381953
BI066844	0.40599119	-1.300479674
ADAMTS17	0.405816801	-1.3010995
CR386967	0.405584855	-1.301924315
BU439835	0.405216702	-1.303234455
CR390105	0.405000781	-1.304003404
CN227294	0.404878097	-1.304440496
CO762907	0.404837885	-1.304583791
CF254707	0.404619728	-1.30536143
CR385503	0.40448299	-1.305849063
PSMA4	0.404413114	-1.306098313
TC226823	0.404196821	-1.306870121
BU110600	0.404017013	-1.307512048
BU106318	0.403536748	-1.309228036
BU472555	0.40308689	-1.310837233
BU129054	0.40295492	-1.311309645
BX934466	0.402894558	-1.311525776
CR354243	0.402773032	-1.311961005
BU340649	0.402523055	-1.312856677
BU220962	0.402400388	-1.313296398
BU357991.1	0.40204598	-1.314567589
BU220799	0.401602852	-1.316158579
TC217917	0.401499308	-1.316530595
CR390962	0.401250603	-1.317424535
BU129623	0.401060254	-1.318109096
D13037	0.400771202	-1.31914925
BX932032	0.400594742	-1.319784611
CN218923.1	0.400289341	-1.320884896

CO760996	0.400222718	-1.321125031
CR389319	0.400068811	-1.321679933
AB061205	0.399879714	-1.322362
DR428079	0.399856812	-1.322444631
AJ720876	0.399854152	-1.322454225
PG-M	0.399679838	-1.323083296
CR386219	0.399618148	-1.323305994
BX540328	0.399536507	-1.323600763
TC191781	0.399512111	-1.323688856
CR523974	0.399504082	-1.32371785
BX273288	0.399289243	-1.32449389
CR389359	0.399090316	-1.325212823
TC209098	0.399083942	-1.325235866
CR407172	0.398958434	-1.32568965
CO763119	0.398849169	-1.326084821
BU421057	0.398806202	-1.326240251
BX935469	0.39842943	-1.327603881
TC205955	0.398171801	-1.328537043
TC225277	0.39807523	-1.328886991
BX932647	0.397947446	-1.329350177
AF082665	0.397698692	-1.330252277
CR391273	0.397336891	-1.331565349
TC224104	0.397306875	-1.331674337
BU250944	0.397209932	-1.332026398
CR733397	0.397200458	-1.332060809
BU455562	0.396850629	-1.333332002
BX935788	0.396648569	-1.33406675
BX931562	0.396625022	-1.3341524
TC200406	0.396229219	-1.335592823
TC211698	0.396054929	-1.336227564
AJ720944	0.395616494	-1.33782552
BU214537	0.395358547	-1.338766483
BX265775	0.395064075	-1.339841434
CR387897	0.395033459	-1.339953243
BU268625	0.394852135	-1.340615605
RUNX1T1	0.394746231	-1.341002604
CR353162	0.394741003	-1.341021711
BX934870	0.394691341	-1.341203226
TC199106	0.394656033	-1.341332293
CR352356	0.394417531	-1.342204418
BU341086.1	0.394305588	-1.342613937

CR406830	0.394103032	-1.343355247
LOC395568	0.393979337	-1.34380813
AJ719443	0.393969491	-1.343844185
D31900	0.393920627	-1.344023133
BU106186	0.393764705	-1.344594293
BU412933	0.393743344	-1.34467256
TC196480	0.393713383	-1.344782341
TC195535	0.393650922	-1.345011237
CV852602	0.393543426	-1.345405254
CR354133	0.393474458	-1.345658108
BU106755	0.393241378	-1.346512962
AJ851433	0.393035367	-1.347268958
TC214641	0.392629075	-1.348761084
CR389766	0.392544232	-1.349072868
TC206921	0.392405032	-1.349584554
CR407450	0.392157578	-1.350494615
TC214156	0.392101247	-1.350701864
BU351732	0.392082492	-1.350770873
X61200	0.392065814	-1.350832243
BU221200	0.391363271	-1.353419727
BX950796	0.390619446	-1.356164325
BU473499	0.389735058	-1.359434383
BX950343	0.389169768	-1.361528454
BU322109	0.388904082	-1.362513716
CO768683	0.388457316	-1.364172012
TC215133	0.388423164	-1.364298856
BU255898	0.388278229	-1.36483728
BU259871	0.387988154	-1.365915489
M60172	0.387898096	-1.3662504
BU226463	0.387889871	-1.366280992
BU214396	0.387756863	-1.366775778
CR385614	0.387683461	-1.367048906
CR389822	0.387628943	-1.367251798
CR406146	0.387561773	-1.367501817
BU126797	0.387431216	-1.367987897
BU288431	0.38735494	-1.368271958
BU351870	0.387102655	-1.369211892
BU235616	0.386963521	-1.369730523
BU473157	0.386899315	-1.369969919
CR388756	0.386784129	-1.370399498
TC220969	0.386674157	-1.370809748

BX931361	0.386576374	-1.371174626
CV891769	0.386527077	-1.371358615
CR391066	0.386300313	-1.37220525
CR390826	0.386200387	-1.372578485
FGD3	0.385756268	-1.374238495
BG710204	0.38540415	-1.375555987
BU445203	0.385324204	-1.375855283
DR429439	0.385288432	-1.375989224
BX932041	0.385248964	-1.376137019
BU273368	0.385108521	-1.376663051
BU380151.1	0.385054666	-1.376864815
HNMT	0.38478873	-1.37786155
CR406402	0.384590292	-1.37860575
COL1A2	0.384267094	-1.379818656
BX935127	0.383655224	-1.382117695
BM440517	0.383609417	-1.382289959
CR523429	0.383581876	-1.382393539
CR353120	0.383328626	-1.383346355
BU229639	0.382888406	-1.385004119
DR428407	0.382671246	-1.385822595
TC220147	0.382634239	-1.385962121
BU473527	0.38226517	-1.38735434
PHOSPHO1	0.381760007	-1.38926212
BX262312	0.38154338	-1.390080999
RBBP7	0.381489416	-1.390285063
BU358753	0.381365455	-1.390753928
LOC422428	0.381353283	-1.390799975
AJ851609	0.380966926	-1.39226234
BU480860	0.380718418	-1.393203731
TC206468	0.380649821	-1.393463696
BU111495	0.380296179	-1.39480465
BARX2	0.379698285	-1.397074613
BU260853	0.379618427	-1.39737807
TC206139	0.379569524	-1.397563934
AJ729923	0.37934162	-1.398430429
CR391764	0.37931682	-1.39852475
BX929845	0.379272872	-1.398691909
BX266246	0.379188003	-1.399014775
LOC423852	0.378984662	-1.399788634
FBN2	0.378678959	-1.400952834
BU303991	0.378569699	-1.401369154

CR390211	0.378274872	-1.402493151
CR406174	0.378254799	-1.402569708
LOC417967	0.377961371	-1.403689302
CR389820	0.37784142	-1.404147234
BU222533	0.377535409	-1.405316135
TC205912	0.377467431	-1.405575925
TC216101	0.377463482	-1.405591018
PRRX2	0.377241386	-1.406440136
DQ683186.1	0.377154034	-1.406774237
CR389330	0.377129813	-1.406866891
TC188625	0.377116435	-1.406918068
BU415383	0.376802117	-1.408121023
TC220401	0.376787735	-1.40817609
TC211846	0.376776837	-1.40821782
AF301551	0.376602631	-1.408885018
BU454030	0.376533343	-1.409150471
LOC419747	0.376295141	-1.410063435
CR385815	0.376173715	-1.410529049
CR352649	0.376158437	-1.410587645
CV858427	0.375878911	-1.411660119
BU265602	0.3756752	-1.412442214
BU484898	0.375617433	-1.412664074
TC203280	0.375287173	-1.413933114
CN236875	0.37527342	-1.413985984
AJ238354	0.375272776	-1.41398846
BX933241.1	0.375171569	-1.414377591
CO761005	0.375157356	-1.41443225
BI392218	0.374989489	-1.415077936
AJ851615	0.374799534	-1.415808935
BU338823	0.374767625	-1.415931766
LOC419316	0.374763226	-1.415948701
TGM2	0.374741773	-1.416031288
TC208086	0.374403796	-1.417333031
AJ719694	0.374317394	-1.417666005
CV855422	0.374202567	-1.418108638
BX262888	0.374088837	-1.418547178
CR523095	0.374041183	-1.418730973
ATP5J2	0.373665998	-1.420178803
BX932292	0.373565987	-1.42056499
TC205426	0.373491864	-1.42085128
CB018359	0.373339865	-1.421438527

CR387690	0.37313207	-1.422241733
BX272691	0.372945894	-1.42296175
AF152358	0.372678243	-1.4239975
TC218824	0.372297428	-1.425472445
CR386697	0.372248871	-1.42566062
EPB41	0.371900901	-1.427009853
AY057941	0.371892749	-1.427041476
AM068168	0.371687243	-1.427838923
TC200484	0.37152772	-1.42845824
TC197025	0.371302616	-1.429332615
BU393026	0.371040684	-1.430350708
BX929804	0.37094309	-1.430730227
CR390118	0.370885405	-1.430954599
BOC	0.37046798	-1.432579242
KCNIP1	0.370018617	-1.434330237
CR386941	0.369629095	-1.435849775
LOC423194	0.369295663	-1.437151776
CR352639	0.36914929	-1.437723712
CO506342	0.369135439	-1.437777843
BU437856	0.369045354	-1.438129968
BU303523	0.368736603	-1.439337462
BG709996.1	0.368645783	-1.439692841
BU274690	0.368632445	-1.439745041
TC219158	0.368585505	-1.439928757
BU139308	0.368506239	-1.440239051
CR405837	0.368246948	-1.441254527
TC205972	0.367535004	-1.444046437
L09550	0.367517985	-1.444113244
CN235382	0.367516538	-1.444118921
AJ719987	0.367454008	-1.444364408
BX934822	0.366448672	-1.448316958
BX932811	0.366063301	-1.44983495
TC216097	0.366060508	-1.449845957
BX932393	0.365851103	-1.450671487
TC216003	0.365595134	-1.451681228
CN228440.1	0.365407633	-1.452421322
DBC1	0.365134146	-1.453501503
CR391215	0.364990394	-1.454069599
LOC416740	0.364816903	-1.454755518
TC202725	0.364733108	-1.455086932
CF253472	0.36472539	-1.45511746

BU256099	0.364612082	-1.455565725
BG711972	0.364574759	-1.455713416
BX950278	0.364485923	-1.456064998
TC217752	0.364354987	-1.456583357
BU447564	0.364118546	-1.457519868
SOD3	0.363841031	-1.458619848
AY040527	0.363630129	-1.459456353
TC200973	0.363526488	-1.459867606
CALB2	0.363493715	-1.459997677
BU199991	0.363291937	-1.46079875
PBX1	0.363273279	-1.460872843
CR406815	0.363260185	-1.460924845
CR386415	0.362570266	-1.463667479
CR389399	0.3624911	-1.463982521
AL584203	0.361847283	-1.466547157
AJ438290	0.361835722	-1.46659325
CR406542	0.361815315	-1.466674617
TC217047	0.361806955	-1.466707955
CR390614	0.361763849	-1.466879849
BU387439	0.361595849	-1.467549979
CR387650	0.361525976	-1.467828784
BU329200	0.361438195	-1.468179124
TC199635	0.361407727	-1.468300743
BM486346.1	0.360978179	-1.470016465
BX931663	0.36078451	-1.470790697
CR386600	0.360632023	-1.471400584
DN928209.1	0.360223132	-1.473037268
BU444777	0.359825863	-1.47462921
TC223243	0.359533858	-1.475800457
BU295381	0.359435396	-1.476195606
BU460005	0.359388961	-1.476382
CR523530	0.359316246	-1.476673928
CV859476	0.359169735	-1.477262306
BX929619	0.35876488	-1.478889426
BU347377	0.358691186	-1.4791858
CR387886	0.358425619	-1.480254333
CR353595	0.358322286	-1.480670319
TC205988	0.358014059	-1.481911851
LOC424448	0.357944818	-1.482190903
CO772891.1	0.357863578	-1.482518374
TC222857	0.357736191	-1.483032015

BU401861.1	0.357726075	-1.483072812
CN229785	0.357608393	-1.4835475
CR386290	0.356557664	-1.487792682
TC189692	0.356355636	-1.488610353
LOC416686	0.356209949	-1.489200284
AJ720622	0.356065582	-1.489785108
BU343640	0.356062369	-1.489798126
U07644	0.355868803	-1.49058263
BU232584	0.355863916	-1.490602441
BU213063	0.355862028	-1.490610096
CR352879	0.3557624	-1.491014054
BU421478	0.355683886	-1.491332478
BU361745	0.355285687	-1.492948526
BX929886	0.355274806	-1.49299271
U46502	0.355254449	-1.493075378
TC221530	0.355103539	-1.493688357
CHRM4	0.354830306	-1.494798861
TC198984	0.354568313	-1.495864483
CR406910	0.354536374	-1.495994444
CR386259	0.354217107	-1.497294206
CTH	0.353853801	-1.498774677
TC189450	0.353820097	-1.498912099
BU237084	0.353806772	-1.498966432
BU392642	0.353709402	-1.499363525
CV856048	0.353706115	-1.499376934
BU470096	0.35355583	-1.499990044
TC211717	0.353397537	-1.500636111
PDZRN3	0.353207933	-1.501410348
CR387376	0.352774966	-1.503179908
CR352632	0.352648708	-1.503696341
CR523041	0.352535205	-1.504160761
CR352470	0.352521157	-1.50421825
BX930242	0.352360833	-1.504874528
CR523624	0.352218075	-1.505459147
BU134865	0.352191657	-1.50556736
TC223162	0.351943836	-1.506582875
CR391193	0.351863592	-1.506911851
SMYD1	0.351550209	-1.508197342
TC224283	0.351529058	-1.508284146
CR389819	0.35143964	-1.50865117
X72378	0.350951188	-1.510657709

BU367571	0.3497752	-1.515500094
TC216837	0.349416635	-1.5169798
CN230128	0.349161891	-1.518031988
CR390006	0.349105468	-1.518265141
TC217288	0.348985958	-1.518759106
TC216637	0.348929988	-1.518990504
BU133577	0.348800589	-1.519525618
AJ851539	0.34848118	-1.520847349
TC227509	0.348253593	-1.521789857
AF272034	0.348199877	-1.5220124
BU370135	0.348159902	-1.522178039
BX934624	0.348102601	-1.5224155
AB052839	0.348086172	-1.522483594
CR391218	0.347620298	-1.524415769
CR389737	0.34651791	-1.528998175
AJ721064	0.346501013	-1.529068526
BU364353	0.346415248	-1.52942566
CR353526	0.346281804	-1.529981515
AF459439	0.346077674	-1.530832223
BU304911	0.345815832	-1.531924175
CR387797	0.345584873	-1.532888025
BX935067	0.344922945	-1.535653994
CO762759	0.344652348	-1.53678625
ZNF236	0.344494449	-1.537447359
BU234331	0.344425156	-1.537737578
CR523607	0.344198592	-1.538686898
TC197104	0.344153587	-1.53887555
BU234140	0.343806306	-1.54033209
CR524415	0.343795043	-1.540379351
CK612399	0.343764337	-1.540508211
CR522884	0.343655501	-1.54096504
CV037738.1	0.343419942	-1.541954276
CR353370	0.34328698	-1.542512953
BU117800	0.343213278	-1.542822728
BU286995.1	0.342722519	-1.544887107
BU339731	0.34225984	-1.546836075
BU382851	0.342119804	-1.547426475
CR388728	0.342038411	-1.547769746
CR391562	0.342028177	-1.547812912
BU329107	0.341920051	-1.548269068
AL587338	0.341903003	-1.548341003

CR524351	0.341587171	-1.549674302
BU459430	0.341123183	-1.55163529
TC205813	0.341067262	-1.551871815
CR353868	0.341065108	-1.551880925
BX934440	0.341053886	-1.551928396
BU434680	0.340947677	-1.55237774
X56930	0.340802751	-1.552991112
AF126963	0.340521395	-1.554182647
BX950827	0.340022848	-1.556296404
BU362713	0.340002829	-1.556381344
BX933946	0.339976265	-1.556494065
CO762290	0.339688071	-1.557717541
TC188003	0.339568975	-1.558223444
BX269390.3	0.339509094	-1.558477876
GDF9	0.33913748	-1.560057863
CR406882	0.339033687	-1.560499465
CR391032	0.339025445	-1.560534539
BU469385	0.33892034	-1.560981875
CR353597	0.33889061	-1.561108431
CR524099	0.338749646	-1.561708657
IFT88	0.338698378	-1.561927018
BX933370	0.338645029	-1.562154275
CR733330	0.338630922	-1.562214376
CR387421	0.338475767	-1.562875548
CN210627.1	0.338423595	-1.563097939
S100B	0.338211671	-1.56400165
CR390997	0.338154788	-1.564244315
BX933712	0.338049371	-1.564694134
BU475739	0.337069051	-1.568883926
CR391178	0.336946448	-1.569408778
TC219925	0.336880205	-1.569692435
XM_415950	0.336702	-1.570455806
CR354267	0.335837439	-1.574165025
CR353293	0.335552627	-1.575389043
Y00789	0.335206293	-1.576878863
CR388705	0.335110279	-1.577292155
CR406367	0.333987451	-1.582134199
BQ038336	0.333566357	-1.583954308
BU361883	0.333555128	-1.584002872
SRL	0.333490189	-1.584283776
DR424648	0.33288134	-1.586920095

BU370502.1	0.332761685	-1.587438766
CN217900	0.332565158	-1.588291064
TC224033	0.332209101	-1.5898365
CR352389	0.331838488	-1.591446868
D31901	0.331689542	-1.592094569
BX933107	0.331529987	-1.592788724
CR385322	0.331497902	-1.592928356
AM066900.1	0.331298683	-1.593795628
BX930427	0.331098039	-1.59466963
U30520	0.330876446	-1.5956355
BQ037514	0.330822107	-1.595872451
BU217290	0.329192425	-1.602996958
CR390367	0.328781425	-1.604799301
BX934362.2	0.328558759	-1.605776692
LOC422791	0.327486031	-1.610494725
BU307434	0.327330435	-1.611180347
CR406589	0.327281814	-1.611394658
CR391393	0.326959604	-1.612815693
TC206752	0.326793026	-1.6135509
X62019	0.326375745	-1.61539425
CR390020	0.32597472	-1.617168011
AY910750	0.325023443	-1.621384317
TC219082	0.32436913	-1.624291571
TC215304	0.324361226	-1.624326726
DN856174	0.324101547	-1.625482187
LOC422830	0.324045896	-1.625729934
X75558	0.324012808	-1.625877251
BX930304	0.324008485	-1.6258965
BU112540	0.323750979	-1.627043539
SYNE1	0.323750607	-1.627045196
BU246221	0.32358861	-1.627767266
CR389451	0.323126289	-1.629829962
TC213036	0.322827151	-1.631166174
CR390135	0.322569224	-1.632319298
BU425530	0.322560184	-1.632359728
BU294251	0.322350054	-1.633299868
SEC24D	0.321800703	-1.635760618
BU315840	0.321534891	-1.636952797
BX933195	0.320836467	-1.640089963
CR389821	0.320348626	-1.642285292
GJA1	0.320330555	-1.642366678

CR353417	0.320296902	-1.64251825
CR523102	0.31997337	-1.643976253
BU222709	0.319952257	-1.64407145
AY329361	0.319943938	-1.644108965
PDCD4	0.319869899	-1.644442858
BX932473	0.31975082	-1.644980034
TC200771	0.319717905	-1.645128552
TC223321	0.319713859	-1.645146812
CN232392	0.319667716	-1.645355043
BU485132	0.319256846	-1.647210539
BU242482	0.319221487	-1.647370332
BU419675	0.318707643	-1.649694476
BU229616	0.318593276	-1.650212275
BX273412	0.318440074	-1.650906192
DR424957.1	0.318162578	-1.652163939
BU287139	0.31815286	-1.652208006
TC224043	0.317572259	-1.6548432
BX932154	0.31754373	-1.654972813
BX266804	0.317374119	-1.655743611
BU279426.1	0.317279711	-1.656172825
TC202485	0.317254797	-1.656286118
CV859074	0.3170858	-1.657054824
CCDC21	0.316900013	-1.657900375
BU393990	0.316630206	-1.659129201
CR522914	0.316242241	-1.660898013
TC216663	0.316110553	-1.661498898
BU214419.1	0.316074609	-1.661662948
CR353636	0.315957359	-1.662198225
BU465437	0.315897986	-1.662469355
AF218056	0.315155486	-1.66586432
BX272472	0.315050243	-1.666346174
TC210875	0.314942326	-1.666840437
DNASE2B	0.314920684	-1.666939577
TC227057	0.314602568	-1.668397646
CR387321	0.31437612	-1.669436461
CR391541	0.31428313	-1.669863261
CR353995	0.31389818	-1.671631429
CR389507	0.31378031	-1.672173271
BU367669	0.313526778	-1.673339429
TC227375	0.312717078	-1.677070085
BX262539	0.312149698	-1.679690027

CR354320	0.311995143	-1.680404525
BU382312	0.311979747	-1.680475721
TC223628	0.311819676	-1.681216128
BU411842	0.311132051	-1.684401073
BU296670	0.311044413	-1.684807504
IL4	0.310839262	-1.685759353
BU259461	0.310724668	-1.686291316
BU108072	0.309880007	-1.690218416
TC214850	0.309611982	-1.691466792
BU350424	0.309181039	-1.69347625
AJ719740	0.309023431	-1.694211864
X17480	0.308719168	-1.695633036
TC225872	0.308541237	-1.696464776
BU440496	0.308224757	-1.697945349
CR386588	0.308114173	-1.698463047
TC202770	0.307995891	-1.699016992
CR390516	0.307879826	-1.69956076
M18746	0.307823122	-1.69982649
CR354277	0.307650384	-1.700636304
TC192129	0.307543504	-1.701137594
CR386696	0.307076799	-1.703328581
CR406714	0.306615722	-1.705496419
BU285805	0.306565392	-1.705733256
BX950327	0.306491009	-1.70608334
CR407007	0.306366132	-1.706671275
BU255242	0.306128075	-1.707792736
CR391484	0.30583342	-1.709182031
BX933488	0.305658007	-1.710009734
TC215733	0.305115243	-1.712573841
CR385857	0.304800365	-1.714063463
CR407410	0.304781982	-1.71415048
BU301975	0.304599433	-1.71501484
AOX1	0.304330901	-1.716287265
BU343432	0.303993534	-1.717887457
TC218935	0.30365781	-1.719481618
CR522994	0.30343324	-1.720548959
CR390143	0.303210756	-1.721607164
CN235251	0.302827169	-1.72343345
AF033670	0.302587921	-1.724573698
BU488504	0.302381526	-1.725558093
CR406264	0.302106114	-1.726872716

CR352395	0.301977466	-1.727487199
TC214761	0.301718462	-1.728725118
CR391404	0.301310554	-1.730676887
CD738497	0.300887664	-1.732703134
BU420964	0.300388321	-1.735099372
TC224793	0.299964558	-1.737136043
BX930430	0.299406964	-1.739820315
BU391712	0.299310059	-1.740287331
RTN1	0.299078761	-1.741402631
BU436641	0.29838846	-1.744736352
CR353981	0.29789476	-1.747125346
BU325999	0.297792756	-1.747619437
TC186546	0.297509393	-1.748992876
TC206120	0.29739822	-1.74953208
BU460515	0.296168704	-1.755508898
BU338496	0.295971572	-1.756469482
BU235647.1	0.295709396	-1.757748012
TC206494	0.29495516	-1.761432446
CR406802	0.294811968	-1.762133001
RCJMB04_18k17	0.29465723	-1.762890429
BU330174	0.294259896	-1.764837161
BU402504	0.293875596	-1.766722533
CR385622	0.292601345	-1.772991691
CR389279	0.292583686	-1.773078765
CR733395	0.291958319	-1.776165673
BX929642	0.291775956	-1.777067094
CR524314	0.291688214	-1.777501
AB082935	0.291519362	-1.77833639
CO420361	0.29141126	-1.778871472
BU482317	0.290989114	-1.780962911
BQ037990	0.290853823	-1.78163383
ENSGALT00000006774.2	0.290848718	-1.781659149
TC198003	0.290792373	-1.781938664
CR385464	0.290707065	-1.782361964
BU438934	0.290610971	-1.782838929
TC204068	0.290377169	-1.784000072
TC222501	0.290193052	-1.784915119
CR352440	0.290157638	-1.785091189
DT659658.1	0.290028243	-1.7857347

CR406297	0.290016511	-1.785793057
BU331465	0.290012453	-1.785813244
BU206540.1	0.28982835	-1.786729375
SMAD9	0.289713617	-1.7873006
TC202691	0.289682972	-1.787453211
BU403218	0.289102891	-1.790345058
BU297778	0.289050661	-1.790605725
CO761841	0.288564279	-1.793035375
TC203837	0.287946491	-1.796127355
BU241296	0.287917328	-1.796273477
J05077	0.287748383	-1.797120274
TC194705	0.287604744	-1.797840624
BU392097	0.28747403	-1.798496466
BU288020	0.287312903	-1.79930531
CR353129	0.28722026	-1.799770579
BU277345	0.286518957	-1.803297499
DR431787.1	0.286296272	-1.804419212
TC189606	0.286211409	-1.804846913
BU478766	0.286001212	-1.805906833
BU331047	0.285891848	-1.806458613
CR733160	0.28527906	-1.809554243
BU283889	0.284829448	-1.811829783
CR388581	0.284802136	-1.811968129
CR405963	0.283944519	-1.81631903
TC200628	0.283828098	-1.816910675
TC223124	0.283375608	-1.819212512
BX929558	0.282946609	-1.82139825
BU370045	0.282568268	-1.823328635
CR391409	0.282567496	-1.823332576
BX934475	0.282365802	-1.824362725
CR353999	0.282159784	-1.825415721
TC203381	0.282159372	-1.825417825
AJ277116	0.281178424	-1.830442199
CR391476	0.280325421	-1.834825514
CR387242	0.280289958	-1.835008037
AJ720374	0.280157277	-1.83569113
CR389610	0.279921534	-1.836905617
AF204170	0.279733692	-1.837874069
TFAP2B	0.279733237	-1.837876412
AJ723821	0.279301953	-1.840102434
CR523537	0.279224347	-1.840503352

DR426476.1	0.279003701	-1.841643835
BX929552	0.278071072	-1.846474425
CR391026	0.277723676	-1.848277925
CR352921	0.277686691	-1.84847006
CR523796	0.277469896	-1.849596837
BU214398	0.277371165	-1.850110278
BX929839	0.276985712	-1.852116537
BU357234	0.276831007	-1.852922551
TC217580	0.276071109	-1.856888178
BU218715	0.275681542	-1.85892542
CR385801	0.275526557	-1.859736716
TC196974	0.275473138	-1.860016451
CR406107	0.274994114	-1.862527356
CR386499	0.274638584	-1.86439377
X56931	0.274257261	-1.866398279
CR524079	0.274083306	-1.867313634
BU278251	0.273903997	-1.868257778
CR388868	0.273497355	-1.870401216
BU250126	0.273448956	-1.870656538
IL15	0.273426145	-1.870776895
CR523482	0.272993264	-1.87306274
NTRK3	0.272772284	-1.874231033
CR386379	0.272693066	-1.874650078
CR352838	0.271738474	-1.879709253
BU225692	0.271192141	-1.882612725
CR388944	0.270601084	-1.885760475
CR405769	0.26996337	-1.889164429
BX274682	0.269637653	-1.89090612
BU350029	0.26940601	-1.892146058
BX934820	0.26858286	-1.896560856
AJ721031	0.267629303	-1.90169201
AB072378	0.267358913	-1.903150321
CXXC5	0.267231622	-1.903837359
BX935106	0.267103247	-1.90453058
TC223929	0.267028111	-1.904936467
CR391147	0.266893001	-1.905666625
CV861853	0.266870917	-1.905786
CR353283	0.266553182	-1.907504692
BU332356	0.265960377	-1.910716768
CO769159	0.265740929	-1.91190765
CR352619	0.265592858	-1.912711741

CR389672	0.265420715	-1.913647123
AF038560	0.265197572	-1.914860529
TC224721	0.264346691	-1.919496825
TC199132	0.264299898	-1.919752225
TC205505	0.263873816	-1.922079893
TC221570	0.263718047	-1.922931795
DR428675.1	0.263648876	-1.92331025
BU427229	0.263289438	-1.925278449
CR733050	0.263247027	-1.925510858
BX929946.1	0.262651102	-1.928780457
BU377399	0.262187879	-1.931327103
AM065402.1	0.261982123	-1.932459724
NP347511	0.261767765	-1.933640644
BX930515	0.261319989	-1.93611061
TC202291	0.260599805	-1.940092091
BU433822	0.260128383	-1.942704275
BX262697	0.259855905	-1.944216254
BU432583	0.259582811	-1.945733242
CR733099	0.259435795	-1.946550552
BU297947	0.25925346	-1.94756485
AF074248	0.25864414	-1.950959589
CV892141.1	0.258414261	-1.952242407
BX932099	0.257722598	-1.956109049
LOC423497	0.2576772	-1.956363207
BU439325	0.257651827	-1.956505275
SLC13A4	0.257025187	-1.960018354
BX935550	0.256999443	-1.960162863
TC217877	0.256828363	-1.96112356
BX265356	0.256518382	-1.962865885
BU374286	0.256303476	-1.964075049
TC201913	0.256274801	-1.964236467
TC223414	0.255985485	-1.965866087
CO767183	0.255767265	-1.967096464
CO767686	0.255463287	-1.968812123
TC195275	0.255287123	-1.969807325
BU408189.1	0.255097321	-1.970880344
CR353974	0.255044971	-1.971176443
BU225561	0.25487684	-1.972127812
CR386305	0.254868715	-1.972173803
ALDH1L2	0.254148368	-1.976257127
BU355829	0.25325426	-1.981341557

BU468610	0.253082578	-1.982319896
CO762725.1	0.252153352	-1.987626691
U17606	0.251845053	-1.989391702
CR406829	0.251233095	-1.992901573
BU263041	0.251126828	-1.993511935
BU225644	0.250880686	-1.994926686
TC219046	0.250783012	-1.99548847
CR386721	0.250709628	-1.995910691
BU418911	0.250431833	-1.997510134
TC226670	0.250140341	-1.999190352
CV891817	0.249463605	-2.003098745
TC220339	0.248997557	-2.005796508
BX931325	0.248826644	-2.006787122
BU410175	0.248817924	-2.006837681
CR523117	0.248720078	-2.00740512
CV854428	0.248561998	-2.008322352
BMPR1B	0.248372644	-2.009421812
AKR1D1	0.247989325	-2.011650076
BU249349	0.247531036	-2.014318672
TC206414	0.246097582	-2.022697612
BX936172	0.245634477	-2.025415027
BU120840	0.245167228	-2.02816195
BU348410	0.244969986	-2.029323098
GREM1	0.244946452	-2.029461699
D26093	0.2445029	-2.03207652
TC214586	0.243160822	-2.040017295
TC211744	0.243145344	-2.040109128
TC213556	0.242524886	-2.043795303
CR390682	0.242295014	-2.045163375
AJ719570	0.242146553	-2.046047628
CR523668	0.241859931	-2.047756319
BU121809	0.241555529	-2.04957322
AJ719339	0.241456484	-2.050164887
CR385996	0.241240115	-2.051458266
CR523896	0.240356376	-2.056753023
CR385201	0.240074549	-2.058445629
AF401291	0.239902767	-2.059478298
BU476481	0.239764751	-2.060308516
STEAP1	0.239693093	-2.060739755
BU469245	0.239426397	-2.062345875
AJ720508	0.238886364	-2.065603591

BU313449	0.238809497	-2.066067882
TC215885	0.238581032	-2.067448749
V00430	0.238348219	-2.06885725
BM487051	0.238313794	-2.069065636
AF070478	0.23674973	-2.078565315
CR389383	0.235985038	-2.083232703
CR354361	0.235793085	-2.084406688
BU445465	0.235050414	-2.088957872
CN237570	0.235027626	-2.08909775
CR353643	0.234616802	-2.091621761
TC190733	0.232827818	-2.102664653
CR353594	0.232109343	-2.107123497
NRXN1	0.23091721	-2.114552395
AJ719623	0.230789929	-2.115347825
CR406502	0.230690329	-2.11597057
BU314853	0.23068188	-2.116023407
AJ720191	0.230427977	-2.117612208
BU328156	0.23024226	-2.118775435
BX933969	0.229963652	-2.12052225
AJ276213	0.229127019	-2.1257805
TC202069	0.228038007	-2.132653794
CR523800	0.227114138	-2.138510574
BX273853	0.227056944	-2.138873936
BU326221	0.226850549	-2.140185942
CR390899	0.226652731	-2.141444547
BU341386	0.226502267	-2.142402608
BU331185	0.226380212	-2.143180239
CR391151	0.226234896	-2.144106615
CR354236	0.226042765	-2.145332353
BU205684	0.225806683	-2.146839911
CR385819	0.225732078	-2.147316647
CR406655	0.224941927	-2.152375504
BU368845	0.224881031	-2.152766123
BU316827	0.224598068	-2.154582577
TC205951	0.224520576	-2.15508043
TC203631	0.224421031	-2.155720218
BX933750	0.224360768	-2.156107666
TRIM47	0.224295774	-2.156525656
CR524035	0.223861801	-2.159319725
CR407473	0.223392588	-2.162346775
LOC420277	0.223368571	-2.162501887

CR352731	0.222938696	-2.165281043
BX933582	0.222455589	-2.16841075
TC192825	0.222239924	-2.169810086
MMP23B	0.222091248	-2.170775555
CR406734	0.220516315	-2.181042695
TC217868	0.220157801	-2.18339013
CR391426	0.219354047	-2.188666771
PEX10	0.219219622	-2.189551159
AF090441	0.219140785	-2.190070084
XM_421086	0.218969878	-2.191195675
BU454663	0.217682511	-2.19970259
CF132825	0.217620897	-2.200110999
DN850556.1	0.217457534	-2.201194402
FGF7	0.217004318	-2.204204346
BX263886	0.215893161	-2.21161055
BU397172	0.214584459	-2.220382498
X59284	0.214382	-2.221744313
CR389229	0.213233424	-2.2294945
BX932159	0.213226337	-2.229542446
AJ851424	0.213122989	-2.230241872
CR353332	0.213039215	-2.230809075
BU131302.1	0.212984153	-2.231182007
BU279604	0.21140582	-2.241913002
BU419630.1	0.209243594	-2.256744641
BX933549	0.208779378	-2.259948875
BU441540	0.20875085	-2.260146022
BU386794	0.208371516	-2.262770017
BU467857	0.207931137	-2.265822283
CR391322	0.207167494	-2.271130443
CR389735	0.206749419	-2.274044819
L36678	0.206052008	-2.27891957
AB100407	0.204811296	-2.287632808
X13874.1	0.204702686	-2.288398065
BX931105	0.204445806	-2.29020963
TC197904	0.204198611	-2.291955044
CN210810	0.203653145	-2.295814
LOC419738	0.203529628	-2.296689273
DR431699	0.203327139	-2.298125306
BU351158	0.202534422	-2.303760968
BX278204	0.202431431	-2.304494786
AJ719936	0.201286313	-2.312679023

TC226798	0.200815974	-2.316054063
HSPB2	0.200704284	-2.31685668
CN779658	0.200702688	-2.316868154
BU449458	0.200546477	-2.31799147
TC202911	0.198445566	-2.333184764
CR523452	0.198301982	-2.334228999
BU304754	0.198266259	-2.334488913
CR523196	0.198037315	-2.3361558
BU214510	0.197515363	-2.339963223
TC217209	0.197243153	-2.341952875
CR353227	0.197026416	-2.343539025
DR425818	0.196711972	-2.34584333
LRRC17	0.196647458	-2.346316555
BU469015	0.196383357	-2.348255425
CDC5L	0.196194341	-2.349644666
CR405850	0.195689362	-2.353362765
BU463476	0.195620856	-2.353867903
LOC429374	0.194646508	-2.361071635
CR386340	0.194433469	-2.362651514
BU246332	0.194357822	-2.363212925
BI392540	0.193554743	-2.369186433
BX936211	0.193124349	-2.372398025
CR353523	0.192328964	-2.378352051
TC227293	0.191659491	-2.38338265
BX929896	0.191302006	-2.38607609
TC195368	0.190292156	-2.393712
CHODL	0.1900912	-2.39523635
CR388826	0.18945392	-2.4000811
CV853817	0.18921018	-2.401938387
TC226125	0.188954939	-2.403885863
TC197267	0.187948595	-2.411589964
BU359818	0.187487566	-2.415133175
CR354044	0.186785263	-2.420547465
RCJMB04_2l15	0.186738298	-2.420910253
BX932039	0.18608642	-2.425955317
CR352859	0.185669246	-2.429193225
CO422300	0.185611638	-2.429640923
BX929401	0.184032854	-2.44196475
ADH1C	0.18317621	-2.44869595
TC196831	0.180180901	-2.472481996
TC204783	0.179647187	-2.47676175

BU389868	0.179597836	-2.477158125
CN221558	0.179127227	-2.480943454
TC189759	0.178328156	-2.487393587
ITGA8	0.176855238	-2.49935915
TC223772	0.176174272	-2.504924842
TC226331	0.174087917	-2.522112026
BU139539	0.173717984	-2.52518098
BX935806	0.173412364	-2.527721334
L40175	0.172645329	-2.534116797
TC223797	0.170351817	-2.553410756
BU246007	0.168371254	-2.57028225
DR425015	0.168302536	-2.570871183
BU455864	0.168287545	-2.570999687
BU352172	0.165785452	-2.59261068
CR386244	0.165108513	-2.598513589
CR387445	0.163317417	-2.614249441
BU274709	0.162456402	-2.621875499
AB021180	0.159795924	-2.645697483
PDLIM3	0.159738288	-2.646217937
CR524268	0.159406466	-2.649217944
BX932351	0.157903169	-2.662887969
AF479650	0.157194466	-2.669377663
CR386695	0.155644646	-2.683672148
BX933888	0.155302966	-2.686842711
TC202369	0.153839371	-2.700503325
SNTB1	0.153187382	-2.706630625
AF093205	0.152836642	-2.709937627
SNW1	0.152294184	-2.71506725
CB017987	0.150953343	-2.727825392
BX935074	0.149589625	-2.740917975
BU392166	0.148686431	-2.749655105
BU413062.1	0.14679533	-2.768122025
BX936125	0.145587399	-2.7800426
CR406117	0.143687796	-2.79899056
CR391304	0.140909079	-2.827163525
BU410124.1	0.14077326	-2.828554778
BU226341	0.14013907	-2.835068864
FBXL5	0.137593483	-2.861515954
X16021	0.137129265	-2.8663916
CR387091	0.137041526	-2.867314975
BU252198	0.136706056	-2.870850935

BU448246	0.136664111	-2.871293664
BX932344	0.136219496	-2.875994898
TC221684	0.135873664	-2.87966225
CR389679	0.135837429	-2.880047042
TC186581	0.135154831	-2.887315017
U90542	0.134305773	-2.896406778
BX930756	0.133667086	-2.90328383
CR386891	0.131255209	-2.929553418
CN222446.1	0.13118177	-2.930360848
BX278129	0.129173108	-2.952622345
ABCA1	0.12858897	-2.9591612
BU408382	0.126792646	-2.979457026
BX933466	0.125747858	-2.991394275
AF139097	0.124616549	-3.004432428
BU202436	0.122517188	-3.02894394
AY156089	0.120539233	-3.05242531
CV861675	0.119103414	-3.069713325
AJ431390	0.11800113	-3.083127421
BI390827.1	0.117879212	-3.084618775
BU392105	0.115179819	-3.118040133
BU229307	0.114634804	-3.124882975
TC203635	0.112873242	-3.147224583
BX932337	0.109650573	-3.18901475
AL584613	0.10883744	-3.199753173
BU387166	0.108804961	-3.20018375
LOC420057	0.107120447	-3.222694204
BU289200	0.105096047	-3.250219689
BX260986	0.103910581	-3.266585525
TC224540	0.101427491	-3.301479363
TC199323	0.100185569	-3.319253381
BU364414	0.099622759	-3.327380825
BU391225	0.099134692	-3.334466175
BX931341	0.094675412	-3.400866394
TC206834	0.092423408	-3.435597902
LOC419240	0.085414687	-3.549372034
TC221684	0.0837279	-3.57814775
TC200970	0.07904775	-3.6611318
XM_415326.2	0.079003408	-3.6619413
BU216363	0.077859533	-3.682982508
BU338989	0.076518448	-3.708048575
TC192961	0.072807933	-3.779760539

BU434933	0.062527239	-3.999371378
AF432506	0.061226719	-4.029694811
BU353997	0.053485627	-4.224704928
FABP4	0.052885989	-4.240970622
BU393904	0.037698373	-4.729353925
TC217234	0.032763398	-4.931771216
BX935789	0.018636736	-5.745706991
CR390488	0.009500848	-6.717727975
